# Nutrient Composition Comparison between a Modified Paleolithic Diet for Multiple Sclerosis and the Recommended Healthy U.S.-Style Eating Pattern

**DOI:** 10.3390/nu11030537

**Published:** 2019-03-01

**Authors:** Catherine A. Chenard, Linda M. Rubenstein, Linda G. Snetselaar, Terry L. Wahls

**Affiliations:** 1 Department of Internal Medicine, University of Iowa, Iowa City, IA 52242, USA; terry-wahls@uiowa.edu; 2 Department of Epidemiology, College of Public Health, University of Iowa, Iowa City, IA 52242, USA; linda-rubenstein@uiowa.edu (L.M.R.); linda-snetselaar@uiowa.edu (L.G.S.)

**Keywords:** Paleolithic diet, exemplary menus, nutritional adequacy, nutrient density, multiple sclerosis, Wahls Elimination diet

## Abstract

Multiple sclerosis (MS) is a demyelinating disease that attacks the central nervous system. Evidence-based dietary guidelines do not exist for MS; the default advice is to follow the Dietary Guidelines for Americans (DGA). A modified Paleolithic Wahls Elimination (WahlsElim) diet promoted for MS excludes grains and dairy and encourages 9+ cups fruits and vegetables (F/V) and saturated fat for cooking. This study evaluated the nutritional adequacy of seven-day menus and modeled them with varying amounts of F/V for comparison with the DGA Healthy US-Style Eating Pattern (HEP) for ages 31–50 years. WahlsElim menus had low added sugar and glycemic index. Nutritional adequacy of the menus and modeled versions were similar to HEP for 17 vitamins and minerals (mean adequacy ratio ≥92%). Nutrient shortfalls for the modeled diet with 60% F/V were identical to HEP for vitamin D, iron (females), magnesium (marginally males), choline and potassium; this modeled diet was also low in dietary fiber and calcium but met vitamin E requirements while HEP did not. WahlsElim-prescribed supplements corrected vitamin D and magnesium shortfalls; careful selection of foods are needed to meet requirements of other shortfall nutrients and reduce saturated fat and sodium. Doctors should monitor nutritional status, supplement doses, and possible contraindications to high vitamin K intake in individuals following the WahlsElim diet.

## 1. Introduction

Multiple sclerosis (MS) is an inflammatory, degenerative neurological disease where the body’s immune system attacks nerves and the myelin sheath. This produces a variety of symptoms including pain, fatigue, and changes in vision, cognition, affect, and movement [1,2]. MS is classified into one of five subtypes based on presentation and disease course: relapsing-remitting (RRMS), primary-progressive, secondary-progressive (SPMS), progressive-relapsing, and clinically isolated syndrome [3,4]. The etiology is still being investigated but genetic, environmental and lifestyle factors are suspected [5]. Although there is no cure for MS, some disease modifying therapies are available [6] and diet and lifestyle changes have shown promising results [7].

The Wahls™ diet was created by Dr. Terry Wahls [8] based on Paleolithic (Paleo) diet guidelines [9,10,11] and a review of the neurodegenerative scientific literature. The Wahls™ diet differs from a traditional Paleo diet in that it exclude eggs; allows legumes (e.g., soy milk) and two servings gluten-free grains (e.g., rice) per week; specifies nine+ cups fruits and vegetables (F/V) per day with one third each from dark-green leafy vegetables, sulfur-rich vegetables, and deeply colored F/V; encourages seaweed, algae and nutritional yeast; limits animal and fish protein [12,13]. The Wahls™ diet was designed to reduce oxidative stress and excitotoxicity [14], nourish the mitochondria [8,15,16], prevent nutrient deficiencies, minimize food allergies and reduce toxic load [16], feed the microbiome, activate anti-inflammatory genes and increase nerve growth factors [8]. Grains [17,18] and dairy [19,20,21] were excluded because of the potential for undesirable immune effects.

A 2009 case report documented Dr. Wahls’ personal use of the Wahls™ diet plus dietary supplements and neuromuscular electrical stimulation (NMES) to treat SPMS which improved mobility from <20 yards (0.9 m) using bilateral support to biking and walking without assistance [14]. A 12-month, non-randomized, single arm pilot study of 20 individuals with progressive MS tested the Wahls™ diet, supplements, NMES, exercise, meditation and self-massage and showed improvements in quality of life (QOL) and fatigue without significant side effects [12,22]. A three-month study of the Wahls™ diet alone in 17 individuals with RRMS also showed significant reduction in fatigue and clinically and statistically significant improvement in QOL in the diet group compared to a wait list control group [23].

Dr. Wahls has continued to refine the dietary guidelines based on current research. The most recent version, Wahls Elimination (WahlsElim), added fermented foods and excluded legumes, nightshades (e.g., white potatoes, eggplant, tomatoes, peppers and their seed spices), and gluten-free grains to further reduce lectin load. Lectins may increase intestinal permeability and immune cell activation [17,18,24]. The dietary guidelines are described in Table 1. A clinical trial of the WahlsElim diet is underway [25,26] and a review of the rationale for this diet was published [27].

PwMS are eager to know what diet(s) will help their disease [28,29] but there is insufficient evidence to support the use of the WahlsElim or any other diet for the treatment of MS including Swank [30], McDougall [31], Mediterranean [32,33], ketogenic [34] or energy restricted/fasting [35,36,37]. Well-designed, randomized controlled trials are needed [38,39] to identify diets that improve disease outcomes. Since studies are not available that provide clear evidence that pwMS have different nutritional needs than the healthy population, the National MS Society (NMSS) [40,41] recommends pwMS follow dietary guidance consistent with the Dietary Guidelines for Americans (DGA) [42] and cancer and heart disease prevention [43,44]. The DGA publishes eating patterns such as the Healthy US-Style Eating Pattern (HEP) [45] that provides serving recommendations for fruit, vegetable, protein food, grain, dairy, and oil consumption at various energy levels which will meet most nutrient requirements [46,47]. However, Dr. Wahls’ work suggests that these guidelines may not produce optimal health in pwMS. It is therefore important to examine the nutritional adequacy of alternative diets for pwMS.

Unlike the HEP, the WahlsElim diet excludes grain and dairy food groups and does not restrict fat or saturated fat. As a result, the WahlsElim diet will not closely adhere to the DGA [42] as assessed by the Healthy Eating Index-2015 (HEI-2015) [48,49]. Although a low HEI-2015 score indicates a lack of adherence to the DGA it does not necessarily indicate the degree of nutritional adequacy. Paleo diets such as WahlsElim that exclude entire food groups are criticized for being unhealthy [50] because it is assumed they will be low in nutrients typically found in the excluded food groups. The NMSS [40] criticized the Paleo diet for potential deficiencies in vitamins B_1_, B_6_, and folate, calcium, vitamin D, and energy. Proponents of Paleo diets, however, disagree and cite the nutrient density [10,11,13,51,52,53] of their eating style. The average nutrient composition for a seven-day 2000 kcal (8368 kJ) Wahls™ diet was published in *The Wahls Protocol: How I Beat Progressive MS Using Paleo Principles and Functional Medicine* [13]. However, the nutrient composition and nutritional adequacy of the WahlsElim diet has not been reported.

Therefore, the purpose of this paper is to report the nutritional adequacy of the WahlsElim diet compared to the Dietary Reference Intakes (DRI) [54,55] for adult men and women. Because the WahlsElim diet has the potential to be low in some nutrients, foods that are significant nutrient contributors will be examined. Nutritional adequacy will be compared with the HEP [45] because the DGA is the default advice for pwMS until there is sufficient scientific evidence to support the adoption of other guidelines. The results presented will identify potential nutrient deficiencies or excesses in the modified Paleo WahlsElim diet.

## 2. Materials and Methods

### 2.1. Study Overview

This study evaluated seven-day Wahls™ menus that were modified to meet WahlsElim diet guidelines. Nutritional adequacy (percent DRIs) for the WahlsElim diet was compared to the nutritional adequacy of the HEP. HEP nutritional adequacy was obtained from publicly available tables [46]. University of Iowa Institutional Review Board oversight was not required because the study did not involve human subjects.

### 2.2. Nutritional Adequacy of Seven-Day WahlsElim Menus

No menus were available that met the WahlsElim diet guidelines. Therefore, seven-day Wahls™ menus, an earlier version of the WahlsElim diet, and associated recipes published in *The Wahls Protocol: How I Beat Progressive MS Using Paleo Principles and Functional Medicine* [13] were modified by a Registered Dietitian (RD) to meet WahlsElim diet guidelines provided by the diet’s author [25,56] (Table 2). Menus are shown in Supplementary Table S1.

An RD calculated the nutrient composition of menus without coffee and tea using Nutrition Data System for Research (NDSR) software version 2017 (Nutrition Coordinating Center (NCC), University of Minnesota, Minneapolis, MN, USA) (May 2017) [57]. The following changes were made to food group counts so they reflected current guidelines [58]: 1. Servings of green leaf lettuce, Bok choy, and parsley were manually re-assigned from the other vegetable category to dark-green vegetables. 2. Reference portion sizes of raw Bok choy and parsley were manually changed from ½ cup to 2 cups. Servings of leafy greens, sulfur-rich vegetables, deeply colored F/V, white F/V, and berries were manually assigned based on WahlsElim diet guidelines. Recipe food group servings were assigned by NDSR based on recipe ingredients.

Nutrient composition of dietary supplements was calculated separately from menus. Nature Made^®^ Multi for Him 50+ (Nature Made Nutritional Products, Mission Hills, CA, USA), the product recommended for the WahlsElim diet, was used for all age and sex categories.

Nutrient composition of the seven-day menus without dietary supplements was proportionately adjusted to produce menus with energy levels appropriate for the varying needs of adult males and females 19 to 70+ years [59]. Nutrient composition with and without dietary supplements was then expressed as a percentage of the Recommended Dietary Allowance (RDA), Adequate Intake (AI), and Tolerable Upper Intake Levels (UL) for the adult age/sex groups [54,55,60]. The average %RDA for 17 vitamins and minerals (vitamins A, C, D, E, B_1_, B_2_, B_3_, B_6_, folate, and B_12_, calcium, copper, iron, magnesium, phosphorus, selenium, and zinc) was calculated in addition to the Mean Adequacy Ratio (MAR) [61] (Equation (1)). The MAR is an average of the %RDAs with values capped at 100% so excesses in some nutrients do not obscure deficiencies of others.

Mean Adequacy Ratio (MAR), % = ∑percent RDA truncated at 100%/number of nutrients.(1)

### 2.3. Nutritional Adequacy of Diets Using Food Pattern Modeling

The nutritional adequacy [46] of the HEP [45] recommended in the DGA [42] was calculated using USDA Food Group nutrient profiles [47,62] and reported by the USDA [46]. The nutrient profiles [62] were developed based on foods consumed in the USA [63] and nutrient composition for forms that were low in fat, added sodium and added sugar [47,64]. The authors requested folate values from the USDA [65] because they were not included with the nutrient profiles.

WahlsElim menus were modeled similar to the HEP using average food group servings on WahlsElim menus and the USDA Food Group nutrient profiles [62]. Using the same nutrient data to calculate WahlsElim model diets and HEP allowed for direct comparison of the two eating patterns and also provided an estimate of the nutritional adequacy of the WahlsElim menus if typical foods consumed in the USA had been used. The WahlsElim menus include very nutrient dense foods (e.g., liver, nutritional yeast) which may not be typically consumed by many individuals in the USA.

The quantity of F/V on the WahlsElim menus exceeds the 95th percentile of USA intake for fruit [66] and total [67] and dark-green vegetables (DGV) [68] which may make compliance difficult. F/V, especially DGV (e.g., broccoli, bok choy, collards, mustard greens, cilantro, parsley, lettuce excluding iceberg, spinach, and kale [58]), are nutrient-dense [69] and their inclusion on the menus will likely impact the nutritional adequacy. Therefore, the nutritional adequacy of two additional WahlsElim diet versions was modeled by reducing F/V subgroup servings proportionately by 30% and 60% to approach more closely the average 95th percentile values for total vegetables and total fruit (2.9 and 2.7 cup equivalents, respectively, for individuals one year and older) while retaining identical amounts of protein foods, oils, solid fats, and added sugar. Nutrient amounts of all modeled diets were factored proportionately to create 1800 and 2200 kcal (7531 and 9205 kJ, respectively) diets before comparing to the DRI for males and females 31–50 years.

### 2.4. Food Sources of Nutrients

WahlsElim menus were examined to determine top food sources of selected nutrients by disaggregating mixtures into their ingredients. Individual foods and recipe ingredients were manually assigned to a food group based on WahlsElim diet categories. The nutrient contribution of each food group was expressed as a percentage of the total amount provided over the seven-day menus and then food groups were ranked in descending order. Food groups contributing <0.05% of total intake were rounded to 0%. Dietary supplements were excluded from calculations.

### 2.5. Data Analysis

The statistician performed descriptive statistics using SAS 9.4 [70] (SAS Institute Inc., Cary, NC, USA) and Microsoft Excel 2010 [71] (Microsoft Corporation, Albuquerque, NM, USA). Seven menu days were used to calculate the average food group servings and nutrient composition. This was a descriptive study so there are no research questions with hypotheses to test statistically. Conclusions were based a comparison of the menu nutrient composition to the DRI [54] or DGA [42] recommendation for that nutrient.

## 3. Results

### 3.1. WahlsElim Menu Composition

Food group data in Table 3 are consistent with WahlsElim diet guidelines with the following minor exceptions: WahlsElim menus provided (a) 6.3 ounces (179 g) organ meat per week, less than the 12 ounces (340 g) encouraged to avoid overstating the amount pwMS are likely to consume; (b) an average of 1.3 tablespoons (11 g) nutritional yeast per day and 0.5 teaspoons (1 g) dried seaweed powder (kelp) per day, slightly above the minimum recommendations; (c) did not contain algae because it was not required. Average daily F/V servings counted using WahlsElim classifications were 3.0 cups leafy greens, 2.2 cups sulfur-vegetables, 5.2 cups deeply colored F/V which included 0.7 cups berries, and 0.3 cup white F/V excluding potatoes. WahlsElim menus were on average low glycemic with zero gluten and an omega 6:3 ratio <4.0 (Table 4).

### 3.2. Nutritional Adequacy

#### 3.2.1. WahlsElim Menus

The MAR score was ≥95% for males and females 19 years and older, indicating nearly all the RDAs were met (Table 5). Average %RDA was >300%. Vitamins B_1_, B_2_, B_3_, B_6_ and B_12_ were in excess of 500% RDA, likely due at least in part to the inclusion of nutritional yeast.

Nutrients below the RDA for females 19 to >70 years and males 51 to >70 years were vitamin D and calcium. No nutrients were below the RDA for males 19–30 and 31–50 years, likely a result of the higher energy level. WahlsElim menus met the AI for the nutrients examined except for linoleic acid (females and males 31 years and older) and choline (females and males 51 years and older). No nutrients were below the AI for females or males 19–30 years, likely due to the higher energy level. Vitamin K levels were ≥888% AI, the highest percentages of all nutrients. WahlsElim menus exceeded the sodium UL for men at all ages and women 19–50 years. Sodium levels were below the UL for females 51–70 and >70 years, likely due to the lower 1600 kcal (6694 kJ) energy for these ages.

WahlsElim menus exceeded the 35% Acceptable Macronutrient Distribution Range (AMDR) [72] upper limit for fat and were below the 45% lower limit for carbohydrate (Table 4); however, they met the 130 gram carbohydrate RDA (Table 5) at these energy levels. WahlsElim menus achieved the recommended <10% of energy from added sugars but did not reach the DGA recommended <10% of energy from saturated fat [42] (Table 4).

#### 3.2.2. WahlsElim Menus Plus Dietary Supplements

When the nutrient contribution from dietary supplements (Table S2) was added to the menus, vitamins D exceeded the RDA. Calcium, linoleic acid, and choline remained below the RDA/AI. All menus exceeded the 100 µg UL for vitamin D and 1000 µg UL for synthetic folate.

#### 3.2.3. Food Pattern Modeling

All WahlsElim modeled diets were lower in carbohydrate and higher in fat than HEP (Table 6). HEP nutritional adequacy was similar to all WahlsElim versions with MAR scores ≥92% and average %RDA 152–210%. All WahlsElim modeled diets were similar to the HEP with low levels of vitamin D, iron (females), and choline; some versions were also low in magnesium (males) and potassium as was HEP. Unlike the HEP, however, one or more WahlsElim modeled diets was low in calcium and dietary fiber. HEP was low in vitamin E but all WahlsElim modeled diets met this RDA.

The addition of dietary supplements to the WahlsElim modeled diets would meet the RDA for vitamin D and magnesium but not the RDA for calcium (males and females) and iron (females) or the AI for choline (males and females); dietary fiber and potassium requirements would also not be met for some WahlsElim modeled diet versions.

Macronutrient composition of the original WahlsElim modeled diet (Table 6) was similar to WahlsElim menus (Table 4) except the percent energy from saturated fat and DHA were higher than menus. Nutrients below the RDA were similar to the menus except the following nutrients were below the RDA on the original modeled diet (Table 6) but not the menus (Table 5): iron (females), vitamin D (males), and calcium (males). The original WahlsElim modeled diet MAR scores were 4 percentage points lower than the menus and the average %RDA was more than 200 percentage points lower. The lower %RDA for vitamin D, calcium and iron on the modeled WahlsElim diet contributed to the reduced MAR. These differences and the >170 percentage point reduction in %RDA values for vitamins A, B_1_, B_2_, B_3_, B_6_, and B_12_ plus copper suggests that foods included on the WahlsElim menus provided higher levels of these nutrients than the aggregate food selections of the USA population which were used to generate the USDA Food Group profiles.

Choline and iron (females) were low in WahlsElim modeled diet but not menus possibly indicating menus contained foods higher in iron and choline than those used to generate USDA Food Group profiles. Conversely, linoleic acid met the AI on the modeled diet but not the WahlsElim menus indicating menu foods may have provided less linoleic acid than foods included in the USDA nutrient profiles. Sodium did not exceed the UL for any of the modeled diets but menus exceeded this limit. Vitamin K again had the highest percentage of all nutrients examined on all the WahlsElim modeled diet versions.

### 3.3. Food Sources of Nutrients on WahlsElim Menus

Food sources of selected nutrients from WahlsElim menus are shown in Supplementary Table S3. Nutrient amounts and food portions reflect the total provided by all seven menus. The colored F/V category was divided into subgroups to investigate the contribution of specific colors. The total of all F/V was reported for energy but was not aggregated for other nutrients.

White and colored F/V were the biggest contributor to energy intake (Table S3A; 30.2%) followed by beef/pork/lamb, nuts and seeds, and coconut milk (total 60.4%). Top fat sources were nuts and seeds, coconut milk and coconut, oil excluding coconut, and beef/pork/lamb (Table S3B; 66.3%). Coconut milk and coconut was the top source of saturated fat (43.5%) followed by coconut oil which together provided 63.4% of the saturated fat (Table S3C). Sulfur vegetables, orange vegetables, leafy greens, and orange fruit contributed 52.1% of the dietary fiber (Table S3E).

For the 24 micronutrients examined, food groups earning the most top spots on the menus were leafy greens (vitamin K, folate, betaine, lutein + zeaxanthin, iron) and nutritional yeast (vitamins B1, B_2_, B_3_, B_6_) followed by sulfur vegetables (vitamin C, calcium, potassium), fish and seafood (vitamin D, vitamin B_12_, copper) and orange vegetables (vitamin A, β-carotene, beta-cryptoxanthin) each with three top spots. Nuts and seeds was the top contributor of vitamin E and magnesium. Organ meat was the top source for retinol, beef/pork/lamb for choline, red fruit for lycopene, and salt for sodium. The following foods also contributed ≥15% of the total amount for these nutrients: leafy greens (vitamin A, beta-carotene and calcium); organ meat (vitamin A, folate, vitamin B_12_, choline, and iron); nutritional yeast (folate and vitamin B_12_); sulfur vegetables (folate); fish and seafood (choline and calcium); orange vegetables (betaine); red vegetables (betaine).

The largest single food contributors to calcium intake among the top three categories on WahlsElim menus were Bok choy, sardines, salmon, and spinach (Table S3X). The majority (83%) of the vitamin D on WahlsElim menus was provided by salmon (Table S3I). Oysters were a significant contributor to copper intake (Table S3Y) with 57 g providing 3.6 times the 0.9 mg copper RDA.

## 4. Discussion

### 4.1. Nutritional Adequacy: Nutrients Above the RDA or AI

WahlsElim menus showed good but not perfect nutritional adequacy with MAR ≥94% that was confirmed by menu modeling and was similar to the HEP. WahlsElim menu average %RDA ranged from 344–496% with levels of vitamins B_1_, B_2_, B_3_, B_6_, B_12_, K, C, and A >400% RDA or AI. Nutritional yeast, fish and seafood, liver, leafy, sulfur, and orange-colored F/V contributed to the high levels of these nutrients on the WahlsElim menus.

Recent articles on nutrient intake of hunter gatherer societies suggest a Paleo diet is more nutrient dense and may exceed the RDAs [11,73]. The average 440% RDA for the 2200 kcal (9205 kJ) WahlsElim menus for 31–50 year old males was similar to a one-day, 2200 kcal (9205 kJ) Paleo menu developed by Cordain [10] that had an average 420% RDA for 14 vitamins and minerals for a 25 year old female. RDAs are established at levels believed to have a low probability of inadequacy for healthy individuals but there are no RDAs specifically for pwMS. Dr. Wahls hypothesizes that intake of certain nutrients at levels above the RDA may be beneficial for myelin repair. Repair of damaged neuronal tissue is likely to require additional structural components beyond what is required for maintenance of healthy tissue. Increased nutrients for the mitochondria may improve mitochondrial bioenergetics and additional omega 3 and omega 6 fatty acids may improve efficiency of remyelination. Traumatic brain injury [74] and burn patients [75] have a higher nutrient need for macro and micronutrients. Thus, pwMS may have higher needs for brain related nutrients.

Vitamin K was the one nutrient consistently in excess of 600% AI on both the WahlsElim menus and all modeled diets. Menu levels of vitamin K (533 µg/1000 kcals; 533 µg/4184 kJ) exceeded the mean (SE) 120.9 (4.68) μg/day average US intake of males and females ≥20 years [76]. The higher amount of vitamin K is largely due to the greater quantity of DGV which exceeds the 95th percentile mean (SE) 0.4 (0.02) cup-eq per day consumed by individuals in the USA [68]. Vitamin K is potentially important for MS because it participates in sphingolipid metabolism, cell membranes, enhances remyelination and oligodendrocyte precursor cells [77,78,79,80]. A recent study found patients with RRMS had a lower vitamin K_2_ level than healthy age matched controls [81]. Vitamin K is believed to have low potential for toxicity but there is insufficient evidence to establish an UL [82]. Vitamin K interacts with some medications such as anticoagulants, antibiotics, bile acid sequestrants, and orlistat [83] so clinicians and pharmacists are advised that pwMS following this diet may have higher than typical vitamin K intake.

Average %RDA on the WahlsElim menus were higher than the modeled diet (387% and 440% versus 182% and 210% for females and males 31–50 years, respectively), indicating menus may contain more nutrient-dense foods than the food group nutrient profiles used to model the diet. One nutrient, iron, was adequate on the WahlsElim menus for females 31–50 years but fell below the RDA on the modeled menus. This change may be partly because liver only contributed 0.21% to the USDA protein food group composite nutrient profile [63] but provided 8.9% of the protein servings on the WahlsElim menus. Liver was the second highest source of iron on WahlsElim menus.

In addition to average %RDA being higher on the menus, the model diets with greater amounts of F/V had higher percentages of vitamins A, C, E, B_1_, B_2_, B_6_, folate, K, copper, magnesium, dietary fiber, manganese, choline, and potassium. Although the quantity of F/Vs on the WahlsElim menus and all model diets is greater than the average US intake [66,67,68], F/V amounts on the WahlsElimA diet were similar to those reported from a pilot study of the Wahls™ diet (7.3–7.8 cups) [22], indicating F/V intake can approach amounts on the WahlsElimA diet with the education, support and accountability provided by a clinical trial. There is some evidence that increased F/V consumption may be beneficial for MS. A case control study of pediatric MS found that a one cup increase in vegetable intake was associated with a 50% reduction in risk for relapse [84].

### 4.2. Nutrients Below the RDA or AI

Nutrients below the RDA or AI varied depending on the food sources (menu versus average US intake) and quantity of F/V. The WahlsElim diet modeled with the most conservative amount of F/V (WahlsElimA) had identical shortfall nutrients as HEP except for calcium and dietary fiber which were low on WahlsElimA but not HEP and vitamin E which was low on HEP but not WahlsElimA.

Most WahlsElim menus and all modeled diets were low in vitamin D, a nutrient of public health concern, as was the HEP. However, the shortfall was corrected by WahlsElim-prescribed supplementation with 125 µg vitamin D. Although this dose exceeds the 100 µg UL it is lower than the 250 µg (10,000 IU) dose recommended to correct low blood levels [85]. In the clinical study evaluating the WahlsElim diet [25], blood levels are measured so supplement dose can be titrated. Target blood level is 40–80 ng/mL based on studies correlating lowest disease activity with blood levels of >40 ng/mL [86]. Individuals following the WahlsElim diet are encouraged to consult with their physician regarding vitamin D dose and have their blood level monitored.

Most WahlsElim menus and all modeled diets were low in calcium unlike the HEP. Calcium is a nutrient of public health concern [42] and requirements can likely not be met from food if dairy products are excluded [87]. Dr. Wahls does not prescribe calcium supplementation based on the advocacy by Jonnson [88] and Cordain [10] who cite various reasons why calcium intake below the RDA may not be detrimental. In addition, there is concern that calcium supplementation may be associated with cardiovascular adverse events, ectopic calcification, and kidney stones [89]. Instead of calcium supplementation, Dr. Wahls advocates supplementation with vitamin D to maintain adequate blood levels and a high intake of vitamin K rich foods primarily from leafy greens. Vitamin D regulates uptake of calcium from the gut and may increase GI absorption of calcium [90]. Gut bacteria metabolize vitamin K into vitamin K2mk7 which facilitates bone mineralization [91,92]. Bone density monitoring may be advised for pwMS following this diet.

Although the WahlsElim menus were not low in iron all modeled diets had low levels of iron for women of childbearing age as did the HEP. The addition of the multivitamin/mineral supplement recommended by Dr. Wahls will not meet the iron RDA for women of childbearing age because a product without iron was selected due to the concern that brain iron contributes to an increased risk of neurodegeneration [93,94]. Therefore, including iron-rich food sources in the diet may be needed to meet the iron RDA for adult women <51 years who have higher iron requirements. Dr. Wahls recommends physicians monitor the complete blood count of pwMS following this diet and respond clinically if there is evidence for microcytosis, insufficient iron stores, or overt anemia.

Some WahlsElim menus and/or model diets were low in choline and potassium as was the HEP. Choline is involved in myelin production [95,96,97] so incorporating good sources such as liver, beef, chicken, and shiitake mushrooms [98] may be advised since the diet may be low in this nutrient. Potassium is another nutrient of public health concern [42]; individuals consuming fewer F/V than the menus may benefit from consumption of higher potassium F/Vs such as beet greens, sweet potato, Swiss chard, spinach, and prunes ([42] Appendix 10) to increase intake of this nutrient.

WahlsElim diets modeled with fewer F/V had levels of dietary fiber below the AI unlike the HEP. F/Vs are the main sources of dietary fiber on the WahlsElim diet other than nuts and seeds. The HEP which contained fewer servings of F/V than the WahlsElimA diet provided adequate amounts of fiber likely because it included fiber-rich grains and legumes which are excluded on the WahlsElim diet. Dietary fiber is a nutrient of public health concern [42] and careful attention to the inclusion of additional fiber sources such as pumpkin and chia seeds, avocado, berries, collards, and prunes ([49] Appendix 13) may be warranted for 31–50 year old females and males consuming less than approximately eight and 12 cup-eq F/V, respectively. In addition, diet, including fermentable and non-fermentable fiber, is a major factor in determining the gut microbiome [99,100,101] which may impact systemic and central nervous system inflammation in MS [102,103].

Most WahlsElim menus were below the AI for linoleic acid, an omega-6 essential fatty acid that forms the phospholipid component of cell membranes. There is insufficient evidence to establish an RDA or UL [104,105] so the optimum amount for health is not known. Dr. Wahls recommends an omega 6:omega 3 ratio of 4:1 to minimize inflammation. The modeled diets were not low in this nutrient suggesting that a more careful selection of food sources on the menus may increase the amount. Replacing coconut milk which is low in linoleic acid with approved cold-pressed oils or nuts containing higher amounts of linoleic acid would increase the amount in the WahlsElim menus.

Missing from this list of nutrients that were below the RDA are the B vitamins. These nutrients are typically thought to be low in Paleo diets because grain products are excluded; however, the WahlsElim menus and all modeled menus met the RDAs for all B vitamins. Thus, the diet pattern is likely to meet the B vitamin RDAs without the use of very rich sources such as nutritional yeast which some individuals may choose not to eat because of taste preferences or adverse symptoms.

### 4.3. Nutrients Outside the AMDR, above DGA Maximum Levels, or above the UL

The NMSS cautioned that Paleo diets such as WahlsElim may be low in energy [40]. Menus in this report were adjusted to provide energy levels estimated for the needs of sedentary individuals. However, several short-term studies reported reduced energy intake and weight loss on a Paleo diet compared to a usual or comparator diet [106,107,108,109]. The increased satiety observed on Paleo diets [88,110] may be a contributor. The energy density of foods and energy-providing beverages on the WahlsElim menus (0.7 kcal/g, 2.9 kJ/g) was lower than the average US diet (1.52 kcal/g, 6.36 kJ/g) [111] and could contribute to weight loss [112,113]. Individuals following the Wahls™ diet as part of a multimodal treatment experienced an average 7.7% reduction in BMI over one year despite being advised to maintain their weight [12]. PwMS who are overweight or obese may benefit from weight reduction associated with the WahlsElim diet. Elevated BMI may be a risk factor for MS [114] and has been associated with increased disability and risk of relapse [115]. However, excessive weight loss as a result of low energy intake could be contraindicated for pwMS with low or underweight BMI who are at risk for malnutrition [116,117,118]. The WahlsElim diet did not provide guidance on food group servings needed to achieve different energy levels. A diet pattern for various energy levels similar to the HEP might help pwMS select quantities of food to maximize nutrient intake and provide appropriate energy to maintain, gain or lose weight as clinically indicated.

The WahlsElim diet will necessarily be lower in carbohydrate and higher in fat that the HEP or DRI recommendations because major carbohydrate sources (grains, dairy, added sugar, legumes) are excluded and protein amounts are moderate. Although the quantity of protein foods on the WahlsElim modeled diets was higher than the HEP, the percent energy from protein was within the AMDR range and was lower than most Paleo diets to minimize the negative effects of mTOR on autoimmunity [119]. Compared to a one-day Paleo menu developed by Cordain [10], WahlsElim menus and modeled diets were lower in protein (20–23% versus 38%) and higher in fat (43–57% versus 39%) and carbohydrate (23–39% versus 23%). Cordain’s 2200 kcal (9205 kJ) Paleo menu was marginally below the 130 gram carbohydrate RDA [10]. WahlsElim menus met the carbohydrate RDA except at energy levels below 1283 kcal (5368 kJ) and for the 1800 kcal (7531 kJ) WahlsElimA modeled diet with the least F/V. Thus, the diet’s energy level and quantity and type of F/V (the main carbohydrate source in a Paleo diet) will impact whether the diet meets the carbohydrate RDA.

Saturated fat levels in the WahlsElim menus and modeled diets exceeded the limit (<10% energy) recommended by the DGA to reduce cardiovascular disease risk [42]. Saturated fats are inflammatory and may also lead to gut dysbiosis [120]. However, Dr. Wahls does not restrict saturated fat unless clinically warranted and prefers animal fats or coconut oil for cooking because they are heat stable [27]. Paleo diets need not be high in saturated fat. Cordain’s menu [10] provided 7% energy from saturated fat using lean meats and nuts and excluding processed oils. The top saturated fat source on WahlsElim menus was coconut milk used in smoothies. Replacing it with water or homemade almond milk and adding lean protein, nuts and/or unsaturated oils to match the energy content would reduce saturated fat so it approaches 10% energy. Clinicians are advised to monitor lipids in pwMS who are following this diet and if adverse laboratory values are noted to recommend the individual decrease saturated fat and increase olive oil intake.

WahlsElim menus exceeded the sodium UL for males ≥19 years and females 19–50 years. However, modeled diets did not exceed the UL because the USDA food group nutrient profiles used to calculate the modeled diets were developed using foods without added sodium [47]. The DRI and DGA recommend reducing sodium intake to <2300 mg per day [42] to reduce risk for high blood pressure. The need to restrict sodium intake specifically for pwMS is controversial and more research is needed [27]. Although the menu sodium level (1350 mg/1000 kcal; 1350 mg/4184 kJ) exceeded the UL at some energy levels it was lower than the mean (SE) sodium intake of US individuals ≥20 years: 4107 (64.1) mg/day for males and 3007 (38.5) mg/day for females [76]. Top food sources of sodium on the menus could be reduced by eliminating salt from recipes, replacing high sodium protein foods such as sausage with lower sodium protein sources, and purchasing low sodium broths. Additional suggestions for reducing sodium levels include selecting fresh or frozen vegetables rather than canned and checking the sodium content on labels of commercial foods.

The diet-prescribed supplemental folate exceeds the UL for this nutrient which was established to prevent neuropathy in individuals who are deficient in vitamin B_12_ [121]; however, it appears unlikely this diet would be low in either vitamin B_12_ or folate based on data in this report. The rationale for the high dose of methylfolate was to reduce the probability of inefficient methylation of folate contributing to elevated homocysteine [122]. Potential adverse effects of high intake of synthetic folate (folic acid) have been reported but with discrepant results and no clear consensus [123]. The WahlsElim diet excludes grains that would be fortified with folic acid in the USA [124] which will reduce additional exposure to synthetic folic acid. However, monitoring homocysteine, vitamin B12, and folate levels to avoid over supplementation is advised.

### 4.4. Limitations

This report did not assess the nutritional adequacy of the WahlsElim diet for children and pregnant or lactating women. Nutrient bioavailability was not considered nor the diet’s inflammatory potential which was associated with MS risk in one study [125]. Levels of iodine [126], sulfur compounds, biotin [127], polyphenols [127] and other components of potential interest for MS were not reported because data were not available.

The authors developed and/or work with the Wahls Protocol^®^ so effort was made to reduce bias favoring the diet. To minimize bias towards the inclusion of nutrient-dense foods like liver and nutritional yeast in the WahlsElim menus, the nutritional adequacy of the diet was estimated by menu modeling using nutrient values for foods typically consumed in the USA. To minimize bias created by the large quantity of F/V in the menus, menus were modeled with reduced amounts of F/V that approached the amounts reported by a pilot study of the Wahls™ diet [12,22].

Menus were only modeled for individuals 31–50 years so additional shortfall nutrients could potentially appear for other ages who have lower energy needs, possibly vitamins E, B1 and magnesium which were minimally above the RDA on the female WahlsElimA diet. Food group nutrient profiles used for menu modeling were generated using foods that were low in fat and added sodium and sugar so the levels of fat, saturated fat and sodium may be underestimated in the modeled diets [128]. Another limitation is the proportionate adjustment of WahlsElim nutrient values to create diets at different energy levels which produces uniform differences in nutrient composition at each energy level, likely a minor limitation.

Adherence [110] to the WahlsElim diet guidelines may be challenging for pwMS, both for the volume of F/V and the unpalatability of some foods such as liver. Individuals may also eat foods that differ from those included on the menus or what was represented by the average of foods consumed in the USA. Thus, the nutritional adequacy of diets selected by pwMS who are following the WahlsElim diet may differ from this report and should be assessed as well as compared to their usual diet. Laboratory data evaluating the nutritional status of individuals following the diet are also needed especially related to lipids, vitamin D, bone, vitamin K, and iron status. Outcome data evaluating the effectiveness of the diet are also needed.

## 5. Conclusions

WahlsElim menus and diets modeled with various quantities of F/V had similar levels of nutritional adequacy for 17 vitamins and minerals as the HEP. WahlsElim supplementation with vitamin D and a multivitamin/mineral without iron corrected low levels of vitamin D and magnesium but not the other shortfall nutrients. Menu modeling of the diets indicate iron may be below the RDA on WahlsElim menus for females <51 years depending on the inclusion of iron rich foods such as liver. The use of nutritional yeast in the WahlsElim menus greatly increased the levels of vitamins B_1_, B_2_, B_3_, B_6_, folate, and B_12_. Vitamin K intake is likely to be increased on this diet due to the recommendation for daily servings of DGV. WahlsElim menus were high in saturated fat due to inclusion of coconut milk. Careful selection of foods may be required to reduce saturated fat, meet calcium, iron, linoleic acid, choline, dietary fiber, and potassium requirements and keep sodium intake below the UL. Studies are needed to evaluate the nutritional adequacy of diets selected by pwMS who are attempting to follow these diet guidelines, assess their nutritional status, and examine the effect of the diet on disease course. At the conclusion of the current clinical study of the WahlsElim diet, participant dietary assessment data and recent MS research will be reviewed to determine any necessary adjustments to the WahlsElim diet and supplementation guidelines.

All pwMS are advised to consult their primary care and neurology team prior to initiating the WahlsElim diet and for monitoring while on the diet. Furthermore, pwMS are strongly advised to continue their disease modifying medications as prescribed and maintain close follow up with their medical team. Abrupt cessation of disease modifying medications increases risk of severe relapse.

## Figures and Tables

**Table 1:** Modified Paleolithic Wahls Elimination [25] diet guidelines.

	Wahls Elimination Diet	
**Saturated Fat**	No restriction	
**Foods Recommended ^3^**	2–3+ cup-eq ^1,2^ (~60–420+ g) dark green leafy vegetables per day	
	2–3+ cup-eq ^1,2^ (~40–765+ g) sulfur-rich vegetables ^4^ per day	
	2–3+ cup-eq ^1,2^ (~40–765+ g) deeply colored fruits/vegetables ^5^ per day	
	6–12+ ounces (170–340 g) meat, fish, poultry, game per day	
**Foods Encouraged ^6^**	12 ounces (340 g) organ meat per week	
	16 ounces (454 g) omega-3 rich fish per week	
	1 serving ^7^ seaweed per day	
	1 serving ^8^ algae per day	
	1 serving ^9^ nutritional yeast per day	
	1 serving ^10^ fermented food per day	
	Clarified butter ^11^, animal fats ^11^, coconut oil/milk ^11^, avocado oil, extra virgin olive oil, sesame oil, sunflower seed oil daily as needed to meet energy needs	
**Foods Limited**	White fruits and vegetables limited each day until leafy, sulfur-rich and colored servings are met ^12^	
	Allowed sweeteners ^13^ ≤1 teaspoon (4–7 g) per day	
	Nuts and seeds, maximum 4 ounces (113 g) per day, soaked preferred	
	Flax, hemp and walnut oil, maximum 2 tablespoons (30 g) per day	
	Alcoholic beverages ≤1 drink per day for women; ≤2 drinks per day for men	
**Foods Not Recommended**	Grains	
	Dairy (cow, goat, mare)	
	Eggs	
	Legumes	
	Nightshade^14^ vegetables/seed spices	
	Non-allowed sweeteners ^15^ or oils ^16^	
	Processed foods	
**Daily Supplements**	1 teaspoon (5 g) cod liver oil	
	1 multivitamin/mineral for men 50+ years	
	1000 µg methyl folate	
	1000 µg methyl B_12_	
	5000 IU (125 µg) vitamin D_3_^17^	

^1^ One cup equivalent = 2 cups raw leafy (~30–140 g), 1 cup raw or cooked (~35–250 g), 1 cup juice (~245–255 g), ½ cup dried (~20–90 g); ^2^ Quantity adjusted depending on appetite and energy needs; ^3^ These foods are core components of the Wahls Elimination diet; adhering to these recommendations is essential to be considered compliant with this diet; ^4^ Includes cruciferous (e.g., broccoli, cauliflower) and allium vegetables (e.g., garlic and onions) as well as mushrooms; ^5^ Includes carrots, beets, cherries, berries, and similar fruits/vegetables with color throughout; a variety of colors including red, blue/black/purple, green, and yellow/orange are encouraged; ^6^ Consumption of these foods is encouraged to enhance nutrient intake of key components believed to be beneficial for MS; ^7^ Serving = ¼ tsp dried powder (0.5 g); ^8^ Serving = 1/2 tsp dried powder (1 g); ^9^ Serving = 1 tablespoon (9 g); ^10^ Serving = ¼ cup fermented vegetable (~80–210 g), ½ cup kombucha tea (122 g); ^11^ Saturated fats are for high heat cooking; ^12^ Includes apples, pears, bananas; allowed after recommended leafy, sulfur and color requirements are met; ^13^ Honey, maple syrup, molasses, sugar; ^14^ tomato, white potato, eggplant, peppers; may be re-introduced to the Wahls Elimination diet after three months if well tolerated; ^15^ Includes artificial sweeteners, sugar alcohols, high fructose sweeteners; ^16^ includes partially hydrogenated fats, margarine, canola oil, soybean oil, corn oil, butter; ^17^ dose adjusted based on blood level.

**Table 2:** Modifications made to Wahls™ menus [13] to comply with Wahls Elimination guidelines.

Modifications
All grains, pseudo grains (i.e., quinoa), and legumes were removed and not replaced Nightshade vegetables tomato, eggplant, white potato and sweet peppers were replaced with equivalent amounts of mango, zucchini, sweet potato, and carrots, respectively. Recipes containing nightshades or other diet non-compliant foods as a major ingredient were replaced with a similar recipe that did not contain the excluded foods. Non-dairy yogurt was removed and not replaced because most commercial products do not meet diet guidelines for added sugar or may contain other ingredients not allowed on the diet. Commercial nut milks were replaced with homemade unsweetened almond milk. Soy milk was replaced with lite (e.g., reduced fat) canned coconut milk when it was a smoothie ingredient and with homemade unsweetened almond milk when it was consumed as a beverage. Six ounces (170 g) ^1^ of cooked liver was added to the seven-day menus. One serving of fermented food (sauerkraut, carrots, beets or kombucha tea) was added daily. Chicken skin was assumed to be eaten. Skillet recipes were individually designed with different spices, liquids, meats and vegetables based on recipes used for the Wahls™ diet calculations; protein sources were assumed to be weighed before cooking, with refuse (i.e., the inedible portion such as bone if one was present in the cut of meat), and no visible fat eaten except for poultry skin.

^1^ Organ meat is encouraged but not required. The menus included half the amount encouraged (12 ounces (340 g)/week; Table 1) to avoid overstating the amount of liver individuals might typically consume.

**Table 3:** Mean food group servings of 1776 kcal (7431 kJ) seven-day modified Paleolithic Wahls Elimination menus.

Food Group	Mean Servings	Food Group	Mean Servings
**Fruits and Vegetables, Total (cup equivalents ^1^)**	**10.3**	**Meat/Fish/Eggs/Nuts/Seeds, Total (servings ^4^)**	**10.1**
**Fruits, Total (cups ^1^)**	3.9	Beef/Pork/Lamb	2.5
Juice	0.1	Poultry	1.3
Whole Fruit	3.9	Fish and Shellfish	2.8
**Vegetables, Total (cup equivalents ^1^)**	**6.4**	Cold Cuts and Sausage	0.4
Dark-green Vegetables	3.3	Organ Meats	0.9
Deep-yellow Vegetables	1.1	Eggs	0.0
Tomato	0.0	Nuts and Seeds including Butters	2.2
White Potatoes	0.0	**Dairy and Nondairy, Total (cup equivalents)**	**0.8**
Other Starchy Vegetables	0.0	Milk, dairy, low fat and fat free	0.0
Other Vegetables	1.9	Yogurt, dairy, fat free	0.0
**Grains, Total (servings ^2^)**	**0.0**	Milk, non-dairy ^5^	0.8
Whole Grain	0.0	**Fats, Total (servings ^6^)**	**5.2**
Some Whole Grain	0.0	Oil	4.8
Refined Grain	0.0	Butter and Other Animal Fats	0.4
**Sweets, Total (servings ^3^)**	**0.4**	Salad Dressing	0.0

^1^ Serving = 2 cups raw green leafy (~30–140 g), 1 cup raw or cooked fruit/vegetable/juice (~35–255 g), ½ cup (~20–90 g) dried fruit; ^2^ Serving = 1 slice bread, ½ cup cooked pasta (70 g), rice (79 g); ^3^ Serving = 4 g sugar, ¼ cup (79 g) syrup, 1 tablespoon (20 g) jam; ^4^ Serving = 1 ounce (28 g) cooked meat/fish/poultry, 1 large whole egg, 2 large egg whites, 1 tablespoon (16 g) peanut butter, ½ ounce (14 g) nuts/seeds; ^5^ Nutrition Data System for Research (NDSR) software counts coconut milk as non-dairy, however, the Dietary Guidelines for Americans (DGA) does not consider it as a dairy serving because it is lower in protein and calcium than cow’s milk; ^6^ Serving = 1 teaspoon (5 g) oil/butter, 15 g mayonnaise.

**Table 4:** Calculated energy, macronutrient, and other components ^1^ for seven-day modified Paleolithic Wahls Elimination menus without dietary supplements.

Nutrient	Mean	SD ^2^	Nutrient	Mean	SD ^2^
Energy (kcal)	1776	154	% Calories from Protein	19.8	3.6
Energy (kJ)	7429	643	% Calories from Fat	42.6	6.0
Total Protein (g)	91.8	11.9	% Calories from SFA	16.4	4.5
Total Carbohydrate (g)	179.8	25.3	% Calories from TRANS	0.2	0.1
Total Dietary Fiber (g)	39.5	4.6	% Calories from MUFA	15.1	1.6
Soluble Dietary Fiber (g)	10.7	2.4	% Calories from PUFA	7.6	2.5
Insoluble Dietary Fiber (g)	28.8	3.8	% Calories from 18:2 linoleic acid ^4^	5.6	1.7
Total Sugars (g)	104.6	14.7	% Calories from 18:3 *n*-3 α-linolenic acid ^4^	1.1	1.2
Added Sugars (by Total Sugars) (g)	2.3	1.4	% Calories from Carbohydrate	37.5	4.3
Gluten (g)	0.0	0.0	% Calories from added sugar ^4^	0.5	0.3
Glycemic Index (glucose reference)	51	8	Total Grams (g)	2572	190
Glycemic Load (glucose reference)	72	19	kcal/Gram	0.7	0.1
Total Fat (g)	87.4	17.7	kJ/Gram	2.9	0.3
Total Saturated Fatty Acids (SFA) (g)	33.8	11.0	Water (g)	2185	189
Total Trans-Fatty Acids (TRANS) (g)	0.5	0.3	sodium:potassium ratio	0.45	0.19
Total Monounsaturated Fatty Acids (MUFA) (g)	30.7	4.8	calcium:phosphorus ratio	0.56	0.1
Total Polyunsaturated Fatty Acids (PUFA) (g)	15.8	5.9	calcium:magnesium ratio	1.84	0.54
Total Conjugated Linoleic Acid (CLA 18:2) (g)	0.1	0.1	Phytic Acid (mg/1000 kcal)	452	135
PUFA 18:2 (linoleic acid) (g)	11.7	4.1	Oxalic Acid (mg/1000 kcal)	439	386
PUFA 18:3 n-3 (alpha-linolenic acid [ALA]) (g)	2.4	2.7	Pantothenic Acid (mg/1000 kcal)	5	2
Omega 6 Fatty Acids (g) ^3^	12.2	4.1	Betaine (mg/1000 kcal)	74	37
Omega-3 Fatty Acids (g)	3.5	2.2			
Omega 6:3 ratio	3.9	1.5			
PUFA 20:5 (eicosapentaenoic acid [EPA]) (g)	0.4	0.4			
PUFA 22:5 (docosapentaenoic acid [DPA]) (g)	0.1	0.1			
PUFA 22:6 (docosahexaenoic acid [DHA]) (g)	0.5	0.4			
Cholesterol (mg)	321	264			

^1^ Calculations were done using multiple decimal places, but results are rounded for display purposes; ^2^ SD = standard deviation; ^3^ Omega-6 Fatty Acids = (PUFA 18:2 (linoleic acid) + PUFA 18:3 (linolenic acid)) – PUFA 18:3 *n*-3 (α-linolenic acid) + PUFA 20:4 (arachidonic acid); ^4^ denominator kcals = (g protein × 4) + (g carbohydrate × 4) + (g fat × 9) + (g alcohol × 7).

**Table 5:** Percent recommended dietary allowance, adequate intake, and tolerable upper intake levels of selected nutrients for the average seven-day modified Paleolithic Wahls Elimination menus without dietary supplements.

	Females				Males			
Age, Years	19–30	31–50	51–70	>70	19–30	31–50	51–70	>70
Energy, kcal	2000	1800	1600	1600	2600	2200	2000	2000
Energy, kJ	8368	7531	6694	6694	10,878	9205	8368	8368
Protein, g	103	93	83	83	134	114	103	103
Carbohydrate, g	202	182	162	162	263	223	202	202
Fat, g	98	89	79	79	128	108	98	98
Saturated Fat, g	38	34	30	30	49	42	38	38
**Percent Recommended Dietary Allowance (RDA)**								
Vitamin A, %RDA	572	515	458	458	579	490	445	445
Vitamin C, %RDA	547	493	438	438	593	502	456	456
Vitamin D, %RDA	**92 ^1^**	**83**	**74**	**55**	119	101	**92**	**69**
Vitamin E, %RDA	132	118	105	105	171	145	132	132
Vitamin B_1_, %RDA	833	750	666	666	993	840	763	763
Vitamin B_2_, %RDA	1017	924	832	832	1017	938	860	860
Vitamin B_3_, %RDA	686	618	549	549	781	661	601	601
Vitamin B_6_, %RDA	826	744	573	573	1074	909	632	632
Folate, %RDA	266	240	213	213	346	293	266	266
Vitamin B_12_, %RDA	797	717	637	637	1035	876	797	797
Calcium, %RDA	**93**	**84**	**62**	**62**	121	102	**93**	**78**
Copper, %RDA	383	345	307	307	498	422	383	383
Iron, %RDA	119	107	214	214	348	295	268	268
Magnesium, %RDA	167	146	129	129	168	136	123	123
Phosphorus, %RDA	239	215	192	192	311	263	239	239
Selenium, %RDA	262	236	210	210	340	288	262	262
Zinc, %RDA	268	242	215	215	254	215	195	195
**Average %RDA**	429	387	345	344	515	440	389	386
**MAR, % ^2^**	99	98	96	95	100	100	99	97
**Percent Adequate Intake (AI)**								
Dietary Fiber, %AI	178	160	169	169	152	129	148	148
Linoleic Acid, %AI	110	**99**	**96**	**96**	101	**85**	**94**	**94**
α-Linolenic Acid, %AI	250	225	200	200	223	189	172	172
Vitamin K, %AI	1184	1065	947	947	1154	977	888	888
Manganese, %AI	344	309	275	275	350	296	269	269
Choline, %AI	118	106	**95**	**95**	119	100	**91**	**91**
Potassium, %AI	127	114	102	102	165	140	127	127
**Percent Tolerable Upper Intake Level (UL)**								
Sodium	**117**	**106**	94	94	**153**	**129**	**117**	**117**

^1^ Bolded values are below Recommended Dietary Allowance or Adequate Intake or above the Tolerable Upper Intake Level; ^2^ MAR = mean adequacy ratio.

**Table 6:** Nutrient composition and nutritional adequacy of Healthy US-Style Eating Pattern ^1^ [46] and Wahls Elimination menus with different total servings of fruits and vegetables ^2,3,4^ for males and females 31–50 years modeled using the United States Department of Agriculture Food Group nutrient profiles.

Category	Females 31–50 Years				Males 31–50 Years			
	HEP ^1^	Wahls ElimA ^2^	Wahls ElimB ^3^	Wahls Elim ^4^	HEP	Wahls ElimA	Wahls ElimB	Wahls Elim
Fruits + Vegetables, cup-eq ^5^/day	4.0	6.1	9.2	11.6	5.0	7.4	11.3	14.2
Fruit, cup-eq/day	1.5	2.3	3.5	4.4	2.0	2.8	4.3	5.4
Vegetable, cup-eq/day	2.5	3.8	5.7	7.2	3.0	4.6	7.0	8.7
Dark-Green, cup-eq/day	0.2	2.0	3.0	3.7	0.3	2.4	3.6	4.6
Protein Foods, ounce-eq ^6^/day	5.0	15.1	13.0	11.5	6.0	18.4	15.9	14.0
Energy, kcals	1797	1800	1800	1800	2198	2200	2200	2200
Energy, kJ	7519	7531	7531	7531	9196	9205	9205	9205
Protein, g	87	104	97	92	100	127	118	112
Protein, %kcal	19	23	22	20	18	23	22	20
Fat, g	61	113	100	90	78	139	122	110
Fat, %kcal	31	**57 ^7^**	**50**	**45 ^3^**	32	**57**	**50**	**45**
Saturated Fat, g	15	31	27	24	20	38	34	30
Saturated Fat, %kcal	8	**16**	**14**	**12**	8	**16**	**14**	**12**
Monounsaturated Fat, %kcal	11	22	20	17	12	22	20	17
Polyunsaturated Fat, %kcal	10	16	14	12	10	16	14	12
EPA ^8^, g	0.1	0.6	0.5	0.5	0.1	0.7	0.6	0.6
DHA ^9^, g	0.2	1.3	1.1	1.0	0.2	1.6	1.3	1.2
Carbohydrate, g	233	**102**	144	177	286	125	177	216
Carbohydrate, %kcal	52	**23**	**32**	**39**	52	**23**	**32**	**39**
Dietary Fiber, g	29	20	28	34	35	25	34	42
**Percent Recommended Dietary Allowance (RDA)**								
Vitamin A, %RDA	125	151	213	259	109	144	202	247
Vitamin C, %RDA	133	276	415	521	141	281	423	531
Vitamin D, %RDA	**45**	**69**	**60**	**53**	**47**	**85**	**73**	**65**
Vitamin E, %RDA	**61**	108	115	121	**74**	132	141	148
Vitamin B_1_, %RDA	153	110	121	129	165	124	135	144
Vitamin B_2_, %RDA	185	118	136	150	175	122	141	155
Vitamin B_3_, %RDA	160	221	210	202	166	236	225	216
Vitamin B_6_, %RDA	274	209	233	251	201	255	284	307
Folate, %RDA	143	116	161	195	172	141	197	239
Vitamin B_12_, %RDA	274	320	277	244	304	392	339	298
Calcium, %RDA	126	**35**	**46**	**55**	134	**43**	**57**	**67**
Copper, %RDA	146	116	149	174	173	142	182	213
Iron, %RDA	**91**	**62**	**73**	**82**	242	170	202	226
Magnesium, %RDA	105	105	123	136	**94**	**98**	114	127
Phosphorus, %RDA	239	182	182	182	266	222	222	223
Selenium, %RDA	193	228	204	186	221	279	250	228
Zinc, %RDA	171	159	155	151	143	141	137	134
**Average %RDA**	154	152	169	182	166	177	196	210
**MAR, % ^10^**	94	92	93	94	95	96	96	96
**Percent Adequate Intake (AI)**								
Dietary Fiber, %AI	114	81	113	137	114	**65**	**91**	110
Linoleic Acid, %AI	143	210	184	165	125	181	159	142
α-Linolenic Acid, %AI	185	254	242	234	157	213	204	196
Vitamin K, %AI	147	703	1045	1306	142	644	958	1197
Manganese, %AI	213	151	191	221	199	145	183	212
Choline, %AI	**77**	**83**	**88**	**93**	**69**	**78**	**83**	**87**
Potassium, %AI	**67**	**74**	**92**	106	**79**	**90**	113	130
**Percent Tolerable Upper Intake Level (UL)**								
Sodium, %UL	75	58	55	52	84	71	67	64

^1^ Healthy US-Style Eating Pattern; ^2^ Wahls Elimination diet with servings of fruits/vegetables reduced by 60%; ^3^ Wahls Elimination diet with servings of fruits/vegetables reduced by 30%; ^4^ Wahls Elimination diet based on 7-day menus, see Table 3; ^5^ cup-equivalents, see Table 3; ^6^ Ounce equivalents, see Table 3; ^7^ Bolded values are outside the Acceptable Macronutrient Distribution Range or below the RDA or AI; ^8^ Eicosapentaenoic acid; ^9^ Docosahexaenoic acid; ^10^ MAR = mean adequacy ratio.

## Supplementary Materials

The following are available online at https://www.mdpi.com/2072-6643/11/3/537/s1, Table S1. Food descriptions and amounts used to calculate the nutrient composition of the seven-day modified Paleolithic Wahls Elimination menus. Table S2. Nutrient composition of dietary supplements prescribed for modified Paleolithic Wahls Elimination diet. Table S3: Food sources of energy and selected nutrients on seven-day modified Paleolithic Wahls Elimination menus.
