# Supplementary material for: Nutrient Composition Comparison between a Modified Paleolithic Diet for Multiple Sclerosis and the Recommended Healthy U.S.-Style Eating Pattern
Source: Nutrients. 2019 Mar 1;11(3):537. doi: 10.3390/nu11030537 (PMC6470485; doi:10.3390/nu11030537)
Supplement: Supplementary file 1 [file nutrients-11-00537-s001.pdf]

Supplementary Materials:

Table S1. Food descriptions and amounts used to calculate the nutrient composition of the seven-day modified Paleolithic Wahls Elimination [1] menus.

Meal	Food Description	Grams[2]	Household Units
----------	Sunday/Day 1	----------	
**Breakfast**	Smoothie		
	kale, raw	16.000	1 CP[3]
	orange, fresh	96.000	1 small - 2 3/8"[4] diameter
	pineapple, fresh	165.000	1 CP, chunks
	coconut, milk, ready-to-drink, canned, lite	240.000	1 CP
	yeast, nutritional, with vitamin B-12	8.813	1 TB[5]
	Wahls Rosemary Chicken with Zucchini		1 serving (1/8 recipe)
	garlic, fresh	2.250	
	rosemary, fresh	0.142	
	chicken, thigh, cooked, skin eaten	66.825	
	squash, zucchini, cooked from fresh	62.625	
	mushrooms, cooked from fresh	37.949	
	carrots, cooked from fresh	16.500	
	vinegar, distilled	3.719	
	oil, coconut	1.703	
	Starwest Botanicals® Organic Kelp powder	0.121	0.0625 TS[6]
	seaweed, kelp - raw	0.4330	
	nutrients, minerals, magnesium	0.0000	
	food industry additives and ingredients, water, lost in evaporation	-0.3160	
	nutrients, minerals, sodium	0.0020	
	nutrients, minerals, potassium	0.0020	
	nutrients, minerals, calcium	0.0000	
	nutrients, minerals, iron	0.0000	
	nutrients, minerals, selenium	0.0000	
	salt, sea	0.375	
**Lunch**	Salad		
	kale, raw	32.000	2 CP
	bok choy, raw	70.000	1 CP
	mango, fresh	82.500	1/2 CP, sliced
	oil, olive	9.000	2 TS
	vinegar, balsamic	31.875	2 TB
	sardines, canned in oil, drained	96.388	4 OZ[7], before draining
	turnip, raw	130.000	1 CP
	peach, fresh	150.000	1 medium - 2 2/3" diameter
	Kombucha Tea	121.550	4 FO[8]
	tea, brewed (from tea leaves or tea bag) - all flavors or plain, regular, unsweetened	118.400	
	sugar, white granulated	3.150	
**Dinner**	Wahls Seafood Stew with Oysters		1 serving (1/4 recipe)
	coconut, milk, ready-to-drink, canned, regular	101.250	
	Wahls Bone Broth	121.488	
	condensed soup, beef broth	46.800	
	water - tap	13.200	
	components for food fortification, minerals, sodium	-0.206	
	components for food fortification, minerals, potassium	0.052	
	condensed soup, chicken broth	30.000	
	water - tap	29.400	
	fats, animal, chicken or turkey	0.420	

Meal	Food Description	Grams[2]	Household Units
	salt, regular	-0.264	
	vinegar, cider	1.867	
	Starwest Botanicals® Organic Kelp powder (see Sunday/Day1 Breakfast)	0.020	0.01 TS
	salt, sea	-0.399	
	mushrooms, cooked from fresh	75.897	
	onion, white, yellow or red, cooked	32.000	
	squash, butternut	60.475	
	Starwest Botanicals® Organic Kelp powder (see Sunday/Day1 Breakfast)	0.486	0.25 TS
	salt, sea	0.750	
	oyster, cooked from fresh or frozen, Eastern	56.699	
	cauliflower, cooked from fresh	48.360	
	coriander leaf, fresh	4.000	
	avocado, black skin - California type	34.000	1/4 each
	Salad		
	lettuce, romaine or cos	141.000	3 CP, chopped
	celery, raw	40.000	1 medium - stalk - 7 1/2" to 8"
	mushrooms, raw	35.000	1/2 CP, sliced
	garlic, fresh	3.000	1 clove
	basil, dried	0.088	1/8 TS
	oil, olive	9.000	2 TS
	vinegar, balsamic	31.875	2 TB
	blackberries, fresh	108.000	3/4 CP
**------------------------------ Monday/Day 2 ------------------------------**			
**Breakfast**	Smoothie		
	parsley, fresh	60.000	1 CP
	grapes, fresh	151.000	1 CP
	kiwi fruit, green	69.000	1 each - 2" diameter
	yeast, nutritional, with vitamin B-12	8.813	1 TB
	water - tap	237.000	1 CP, without ice
	molasses, blackstrap	7.021	1 TS
	raisins, uncooked	48.333	1/3 CP
	walnuts	14.625	2 TB, chopped
	Homemade Almond Milk, Unsweetened Vanilla	120.030	1/2 CP
	water, tap	116.500	
	nuts and seeds, almonds, raw (dried - unblanched)	3.050	
	ingredient, vanilla extract	0.250	
	spices, salt, regular	0.231	
**Lunch**	Wahls Skillet: Ham and Collards		1 serving (1/4 recipe)
	oil, coconut	3.406	
	garlic, fresh	3.000	
	vinegar, balsamic	5.977	
	Starwest Botanicals® Organic Kelp powder (see Sunday/Day1 Breakfast)	0.486	0.25 TS
	salt, sea	1.500	
	pork, ham, regular cured, boneless, regular, regular, no visible fat eaten	127.573	
	pork, bacon, regular, regular cut, regular	7.938	
	collards, cooked from fresh	70.538	
	sweet potato, baked	176.000	1 CP, chopped, after cooking
	oil, olive	4.500	1 TS
	cinnamon (ground)	0.325	1/8 TS
	raspberries, fresh, red	123.000	1 CP, whole pieces
	Homemade Almond Milk, Unsweetened Vanilla (see	360.093	1.5 CP

Meal	Food Description	Grams[2]	Household Units
	Monday/Day 2 Breakfast)		
	sauerkraut	59.000	1/4 CP
**Dinner**	Wahls Skillet: Lamb Chops and Broccoli		1 serving (1/4 recipe)
	oil, coconut	3.406	
	rosemary, fresh	0.213	
	vinegar, balsamic	5.977	
	Starwest Botanicals® Organic Kelp powder (see Sunday/Day1 Breakfast)	0.486	0.25 TS
	salt, sea	1.500	
	lamb, chops, loin, no visible fat eaten	67.631	
	broccoli, cooked from fresh	144.788	
	Salad		
	spinach, raw	120.000	4 CP
	orange, fresh	90.000	1/2 CP, sections
	mushrooms, raw	17.500	1/4 CP, sliced
	onion, white, yellow or red, raw	40.000	1/4 CP, chopped
	oil, olive	13.500	1 TB
	vinegar, balsamic	31.875	2 TB
	apple, fresh, with skin	182.000	1 medium - 3" diameter
	Wahls Fudge		1 serving (1/20 recipe)
	oil, coconut	10.900	
	raisins, uncooked	7.250	
	nuts and seeds, walnuts	5.000	
	avocado, black skin - California type	6.800	
	coconut, dried (shredded or flaked), unsweetened	2.000	
	cinnamon (ground)	0.195	
	cocoa powder, unsweetened	0.090	
**---------- Tuesday/Day 3 ----------**			
**Breakfast**	Smoothie		
	spinach, raw	30.000	1 CP
	honeydew melon, fresh	170.000	1 CP, cubed
	kiwi fruit, green	69.000	1 each - 2" diameter
	Homemade Almond Milk, Unsweetened Vanilla (see Monday/Day2 Breakfast)	240.062	1 CP
	yeast, nutritional, with vitamin B-12	8.813	1 TB
	Wahls Skillet: Lamb Chops and Broccoli		1 serving (1/4 recipe)
	oil, coconut	3.406	
	rosemary, fresh	0.213	
	vinegar, balsamic	5.977	
	Starwest Botanicals® Organic Kelp powder (see Sunday/Day1 Breakfast)	0.486	0.25 TS
	salt, sea	1.500	
	lamb, chops, loin, no visible fat eaten	67.631	
	broccoli, cooked from fresh	144.788	
	carrots, raw	61.000	1/2 CP, sliced
	dates	42.600	6 each
**Lunch**	Wahls Salmon Salad		1 serving (1/3 recipe)
	salmon, canned - drained, pink, with salt	113.006	
	onion, white, yellow or red, raw	11.667	
	garlic, fresh	1.000	
	celery, raw	8.417	
	parsley, fresh	5.000	
	lime juice, canned, bottled, or boxed (substitute for peanut sauce)	10.250	
	Starwest Botanicals® Organic Kelp powder (see Sunday/Day1 Breakfast)	0.216	0.11 TS

Meal	Food Description	Grams[2]	Household Units
	salt, sea	0.500	
	Salad		
	spinach, raw	105.000	3.5 CP
	raspberries, fresh, red	61.500	1/2 CP, whole pieces
	zucchini, raw	56.500	1/2 CP, sliced
	oil, olive	9.000	2 TS
	vinegar, balsamic	31.875	2 TB
	watermelon, fresh	152.000	1 CP, cubed
	honeydew melon, fresh	170.000	1 CP, cubed
	Homemade Almond Milk, Unsweetened Vanilla (see Monday/Day 2 Breakfast)	240.062	1 CP
**Dinner**	Wahls Skillet: Steak with Mustard Greens and Mushrooms		1 serving (1/4 recipe)
	oil, coconut	3.406	
	garlic, fresh	3.000	
	vinegar, balsamic	5.977	
	Starwest Botanicals® Organic Kelp powder (see Sunday/Day1 Breakfast)	0.486	0.25 TS
	salt, sea	1.500	
	beef, steak - beef, t-bone, no visible fat eaten	93.690	
	mushrooms, cooked from fresh	21.158	
	mustard greens, cooked from fresh	61.250	
	squash, winter types (dark green or orange), cooked	205.000	1 CP, cubed, after cooking
	butter, clarified butter or butter oil	8.542	2 TS
	Wahls Fudge (see Monday/Day 2 Dinner)	32.235	1 serving (1/20 recipe)
	pear, fresh	178.000	1 medium - 2 1/2" diameter
	Homemade Almond Milk, Unsweetened Vanilla (see Monday/Day 2 Breakfast)	240.062	1 CP
	sauerkraut	59.000	1/4 CP
--- **Wednesday/Day 4** ---			
**Breakfast**	Smoothie		
	kale, raw	16.000	1 CP
	orange, fresh	96.000	1 small - 2 3/8" diameter
	coconut, milk, ready-to-drink, canned, lite	240.000	1 CP
	yeast, nutritional, with vitamin B-12	8.813	1 TB
	almonds, raw (dried - unblanched)	42.524	1.5 OZ, edible portion
	apricot, dried, uncooked	21.000	6 half
	prune, dried, uncooked	38.000	4 each
**Lunch**	Wahls Liver Pate		1 serving (1/4 recipe)
	pork, bacon, regular, regular cut, regular	7.938	
	mushrooms, cooked from fresh	18.974	
	Starwest Botanicals® Organic Kelp powder (see Sunday/Day1 Breakfast)	0.243	0.125 TS
	onion, white, yellow or red, cooked	43.375	
	poultry, chicken, liver	58.590	
	vinegar, balsamic	1.992	
	salt, sea	0.375	
	oil, olive	4.500	
	oil, coconut	4.542	
	butter, clarified butter or butter oil	4.271	
	vinegar, balsamic	7.969	
	vinegar, cider	7.469	
	carrots, raw	61.000	1 medium - 6" to 7" long
	grapes, fresh	151.000	1 CP
	Homemade Almond Milk, Unsweetened Vanilla (see Monday/Day 2 Breakfast)	480.124	2 CP
	Fermented Beets	72.454	4 TB

Meal	Food Description	Grams[2]	Household Units
	beets, raw	70.654	
	salt, sea	1.800	
**Dinner**	Wahls Coconut Milk Fish Soup		1 serving (1/4 recipe)
	Wahls Bone Broth (See Monday/Day 1 Dinner)	364.465	
	coconut, milk, ready-to-drink, canned, regular	101.250	
	Starwest Botanicals® Organic Kelp powder (see Sunday/Day1 Breakfast)	0.486	0.25 TS
	salt, sea	0.750	
	Chinese cabbage, Pak-choi, cooked	85.000	
	broccoli, cooked from fresh	64.350	
	carrots, cooked from fresh	14.000	
	mushrooms, shiitake, raw	56.699	
	ginger root - raw	1.500	
	salmon, cooked from fresh or frozen, chinook (king)	112.774	
	lime juice, fresh	7.563	
	sweet potato, baked	88.000	1/2 CP, chopped, after cooking
	Salad		
	lettuce, romaine or cos	164.500	3.5 CP, chopped
	cilantro, fresh	8.000	1/2 CP
	garlic, fresh	3.000	1 clove
	oil, olive	9.000	2 TS
	lime juice, fresh	30.250	2 TB
	strawberries, fresh	152.000	1 CP, halves
	banana, fresh or ripe	75.000	1/2 CP, sliced
-- **Thursday/Day 5** --			
**Breakfast**	Smoothie		
	beets, raw	68.000	1/2 CP, whole pieces
	mango, fresh	82.500	1/2 CP, sliced
	blueberries, fresh	74.000	1/2 CP
	coconut, milk, ready-to-drink, canned, lite	240.000	1 CP
	ginger root - raw	1.000	1/2 TS, sliced
	yeast, nutritional, with vitamin B-12	8.813	1 TB
	sardines, canned in oil, drained	92.000	1 can - each 3 3/4 OZ net weight
	celery, raw	50.500	1/2 CP
**Lunch**	Wahls Liver, Onions, and Mushrooms		1 serving (1/4 recipe)
	pork, bacon, regular, regular cut, regular	15.876	
	mushrooms, cooked from fresh	37.949	
	Starwest Botanicals® Organic Kelp powder (see Sunday/Day1 Breakfast)	0.486	0.25 TS
	onion, white, yellow or red, cooked	86.750	
	poultry, chicken, liver	117.180	
	vinegar, balsamic	3.984	
	salt, sea	0.750	
	Salad		
	bok choy, raw	210.000	3 CP
	squash, summer type (green or yellow), raw	56.500	1/2 CP, sliced
	garlic, fresh	3.000	1 clove
	oil, olive	9.000	2 TS
	lime juice, fresh	30.250	2 TB
	plum, fresh	132.000	2 medium - 2 1/8" diameter
	Homemade Almond Milk, Unsweetened Vanilla (see Monday/Day 2 Breakfast)	240.062	1 CP
**Dinner**	Wahls Rosemary Chicken with Zucchini		1 serving (1/8 recipe)
	garlic, fresh	2.25	

Meal	Food Description	Grams[2]	Household Units
	rosemary, fresh	0.142	
	poultry, chicken, thigh, skin eaten	66.825	
	squash, zucchini, cooked from fresh	62.625	
	mushrooms, cooked from fresh	37.949	
	carrots, cooked from fresh	16.5	
	vinegar, distilled	3.719	
	oil, coconut	1.703	
	Starwest Botanicals® Organic Kelp powder (see Sunday/Day1 Breakfast)	0.121	0.0625 TS
	salt, sea	0.375	
	squash, butternut	240.000	1 CP, mashed
	Salad		
	bok choy, raw	210.000	3 CP
	carrots, raw	61.000	1/2 CP, sliced
	cucumber, raw, with peel	52.000	1/2 CP, sliced
	grapes, fresh	75.500	1/2 CP
	garlic, fresh	3.000	1 clove
	sunflower butter, without salt	16.000	3 TS
	lime juice, fresh	30.250	2 TB
	peach, fresh	154.000	1 CP, sliced
	almonds, raw (dried - unblanched)	11.500	2 TB, sliced
	Kombucha Tea (see Sunday/Day 1 Lunch)	121.550	4 FO
**-------------------- Friday/Day 6 --------------------**			
**Breakfast**	Smoothie		
	cilantro, fresh	16.000	1 CP
	orange, fresh	96.000	1 small - 2 3/8" diameter
	pineapple, fresh	165.000	1 CP, chunks
	yeast, nutritional, with vitamin B-12	8.813	1 TB
	water - tap	237.000	1 CP, without ice
	grapefruit, fresh, pink or red	115.000	1/2 CP, sections
	Homemade Almond Milk, Unsweetened Vanilla (see Monday/Day 2 Breakfast)	240.062	1 CP
**Lunch**	Salad		
	bok choy, raw	210.000	3 CP
	lettuce, romaine or cos	94.000	2 CP, chopped
	avocado, black skin - California type	37.500	1/4 CP, cut pieces
	mango, fresh	82.500	1/2 CP, sliced
	carrots, raw	61.000	1/2 CP, sliced
	mushrooms, raw	35.000	1/2 CP, sliced
	almonds, raw (dried - unblanched)	13.500	2 TB, slivered
	oil, olive	9.000	2 TS
	lime juice, fresh	30.250	2 TB
	chicken, breast, skin eaten	130.000	1 medium - split
	banana, fresh or ripe	118.000	1 medium - 7" to 7 7/8" long
	grapes, fresh	75.500	1/2 CP
	Kombucha Tea (see Sunday/Day 1 Lunch)	121.550	4 FO
**Dinner**	Wahls Skillet: Salmon, Bacon, Mustard Greens, Onions		1 serving (1/4 recipe)
	oil, coconut	3.406	
	ginger root - raw	3.000	
	lime juice, fresh	5.672	
	Starwest Botanicals® Organic Kelp powder (see Sunday/Day1 Breakfast)	0.121	0.0625 TS
	salt, sea	1.500	
	salmon, cooked from fresh or frozen, coho (silver), wild	86.750	
	pork, bacon, regular, regular cut, regular	15.876	
	onion, white, yellow or red, cooked	34.125	

Meal	Food Description	Grams[2]	Household Units
	mustard greens, cooked from fresh	70.438	
	Wahls Mashed Turnips		1 serving (1/4 recipe)
	turnip, cooked	87.260	
	yeast, nutritional, with vitamin B-12	17.625	
	chives, raw	3.000	
	garlic, fresh	3.000	
	oil, coconut	6.813	
	Starwest Botanicals® Organic Kelp powder (see Sunday/Day1 Breakfast)	0.121	0.0625 TS
	salt, sea	0.375	
	pepper (ground), black	0.144	
	oil, olive	4.500	1 TS
	peach, fresh	150.000	1 medium - 2 2/3" diameter
	cherries, fresh, sweet	77.000	1/2 CP
	Homemade Almond Milk, Unsweetened Vanilla (see Monday/Day 2 Breakfast)	240.062	1 CP
-- **Saturday/Day 7** --			
**Breakfast**	Smoothie		
	collards, raw	36.000	1 CP
	watermelon, fresh	76.000	1/2 CP, cubed
	cantaloupe, fresh	80.000	1/2 CP, cubed
	coconut, milk, ready-to-drink, canned, lite	240.000	1 CP
	yeast, nutritional, with vitamin B-12	8.813	1 TB
	cherries, fresh, sweet	77.000	1/2 CP
	walnuts	29.250	4 TB, chopped
**Lunch**	Wahls Kale Sausage Soup		1 serving (1/4 recipe)
	Wahls Bone Broth (See Monday/Day 1 Dinner)	485.953	
	kale, cooked from fresh	18.200	
	yams (sweet potato type), boiled	66.880	
	onion, white, yellow or red, cooked	34.125	
	Starwest Botanicals® Organic Kelp powder (see Sunday/Day1 Breakfast)	0.486	0.25 TS
	salt, sea	0.750	
	sausage, bratwurst, regular, regular, regular	75.000	
	coconut, milk, ready-to-drink, canned, regular	101.250	
	Salad		
	spinach, raw	60.000	2 CP
	kale, raw	32.000	2 CP
	cauliflower, raw	53.500	1/2 CP
	garlic, fresh	3.000	1 clove
	mushrooms, raw	35.000	1/2 CP, sliced
	almonds, raw (dried - unblanched)	16.250	2 TB, chopped
	oil, flax seed	9.083	2 TS
	vinegar, cider	29.875	2 TB
	pineapple, fresh	165.000	1 CP, chunks
**Dinner**	Wahls Skillet: Pork Chops and Red Cabbage		1 serving (1/4 recipe)
	oil, coconut	3.406	
	garlic, fresh	3.000	
	vinegar, red wine	5.602	
	Starwest Botanicals® Organic Kelp powder (see Sunday/Day1 Breakfast)	0.486	0.25 TS
	salt, sea	1.500	
	pork, chops, fresh, loin, no visible fat eaten	91.852	
	cabbage, red, cooked	113.906	
	sweet potato, baked	132.000	3/4 CP, chopped, after cooking

Meal	Food Description	Grams[2]	Household Units
	oil, olive	4.500	1 TS
	asparagus, cooked from fresh	90.000	6 medium - spear - 5 1/4" to 7"
	blueberries, fresh	148.000	1 CP
	fermented carrots	27.900	1/4 CP
	carrots, raw	27.500	
	salt, sea	0.400	

[1] adapted from the Wahls™ menus and recipes published in: Wahls T, Adamson E. *The Wahls Protocol: How I Beat Progressive MS Using Paleo Principles and Functional Medicine*. New York: Avery; 2014; [2] weights from the Minnesota Nutrition Data System for Research *NDSR* [computer program]. Version 2017. Minneapolis, MN, USA: Nutrition Coordinating Center; 2017. Values are unrounded and are not meant to imply this level of accuracy. Weights for cooked foods are the weight after cooking, edible portion. [3] CP = cup; [4] " = inches; [5] TB = tablespoon; [6] TS = teaspoon; [7] OZ = ounce; [8] FO = fluid ounce.

Table S2. Nutrient composition[1] of dietary supplements prescribed for modified Paleolithic Wahls Elimination diets.

Nutrient	WahlsElim[2,3] Males & Females ≥19 years
Energy (kcal)	45
Total Protein (g)	0
Total Fat (g)	5
Total Carbohydrate (g)	0
Total Saturated Fatty Acids (SFA) (g)	1
Total Monounsaturated Fatty Acids (MUFA) (g)	0
Total Polyunsaturated Fatty Acids (PUFA) (g)	0
Omega-3 Fatty Acids in Supplements (mg)	1100
Cholesterol (mg)	20
PUFA 20:5 (eicosapentaenoic acid [EPA]) (g)	0.4
PUFA 22:6 (docosahexaenoic acid [DHA]) (g)	0.5
Total Vitamin A Activity (International Units) (IU)	3350
Vitamin D (calciferol) (mcg)	**160**[4]
Vitamin E (International Units) (IU)	70
Vitamin K (phylloquinone) (mcg)	80
Vitamin C (ascorbic acid) (mg)	180
Thiamin (vitamin B1) (mg)	1.5
Riboflavin (vitamin B2) (mg)	1.7
Niacin (vitamin B3) (mg)	20
Pantothenic Acid (mg)	10
Vitamin B-6 (mg)	6
Synthetic Folate (folic acid) (mcg)	**1400**[5]
Vitamin B-12 (cobalamin) (mcg)	1025
Biotin (mcg)	30
Lycopene (mcg)	0
Calcium (mg)	162
Chromium (mcg)	120
Copper (mg)	2
Iodine (mcg)	150
Iron (mg)	0
Magnesium (mg)	100

Nutrient	WahlsElim[2,3] Males & Females ≥19 years
Manganese (mg)	4
Molybdenum (mcg)	75
Phosphorus (mg)	0
Potassium (mg)	0
Selenium (mcg)	105
Sodium (mg)	0
Zinc (mg)	15
Total Dietary Fiber (g)	0
Soluble Dietary Fiber (g)	0
Insoluble Dietary Fiber (g)	0
Betaine (mg)	0
Choline (mg)	0
Caffeine (mg)	0
Phytic Acid (mg)	0
Total Trans-Fatty Acids (TRANS) (g)	0
PUFA 18:3 n-3 (alpha-linolenic acid [ALA]) (g)	0

[1] Obtained from Nutrition Data System for Research (NDSR) software version 2017, developed by the Nutrition Coordinating Center (NCC), University of Minnesota, Minneapolis, Minnesota, USA (May 2017); [2] Wahls Elimination menu; [3] all individuals participating in the clinical study comparing the Swank and Wahls Elimination diets (www.clinicaltrials.gov NCT02914964) will receive these supplements regardless of the diet to which they are randomized; [4] exceeds Tolerable Upper Intake Level of 100 mcg; [5] exceeds Tolerable Upper Intake Level of 1000 mcg.

Table S3. Food sources of energy and selected nutrients[1] on seven-day modified Paleolithic Wahls Elimination menus.

**Table S3A. Energy (7-day total 12,429 kcal)**				
**Rank**	**Food Group**	**Total kcal**	**% Total**	**Cumulative %**
**1**	**Beef/Pork/Lamb**	**1344**	**10.8**	**10.8**
**2**	**Nuts and seeds**	**1289**	**10.4**	**21.2**
**3**	**Coconut milk and coconut**	**1116**	**9**	**30.2**
4	Fish and seafood	987	7.9	38.1
5	Fats, oil excluding coconut	836	6.7	44.8
**6**	**Color-Orange-Fruit**	**829**	**6.7**	**51.5**
**7**	**Color-Orange-Vegetable**	**740**	**6**	**57.4**
**8**	**Color-Green-Fruit**	**697**	**5.6**	**63.1**
9	Sulfur	662	5.3	68.4
**10**	**Color-Blue-Fruit**	**633**	**5.1**	**73.5**
11	Poultry	559	4.5	78
12	Fats, oil, coconut	508	4.1	82.1
**13**	**Color-White-Fruit**	**368**	**3**	**85**
**14**	**Color-Red-Fruit**	**358**	**2.9**	**87.9**
15	Nutritional yeast with vitamin B12	297	2.4	90.3
16	Organ Meat	294	2.4	92.7
17	Leafy	291	2.3	95
18	Vinegar	159	1.3	96.3
19	Broth	146	1.2	97.5

Table S3A. Energy (7-day total 12,429 kcal)				
**Rank**	**Food Group**	**Total kcal**	**% Total**	**Cumulative %**
20	Clarified Butter and Animal Fat	112	0.9	98.4
**21**	**Color-Red-Vegetable**	**60**	**0.5**	**98.8**
22	Sweeteners	57	0.5	99.3
**23**	**Color-Green-Vegetable**	**52**	**0.4**	**99.7**
24	Ingredients (e.g., vanilla, baking soda, cocoa)	15	0.1	99.8
25	Kelp, dried powder	10	0.1	99.9
26	Spices	8	0.1	100
27	Tea	4	0	100

Table S3B: Total Fat (7-day total 611.9 g fat)				
**Rank**	**Food Group**	**Total g**	**% Total**	**Cumulative %**
**1**	**Nuts and seeds**	**116.3**	**19.0**	**19.0**
**2**	**Coconut milk and coconut**	**116.0**	**18.9**	**37.9**
**3**	**Fats, oil excluding coconut**	**94.6**	**15.5**	**53.4**
**4**	**Beef/Pork/Lamb**	**79.1**	**12.9**	**66.3**
5	Fats, oil, coconut	56.5	9.2	75.6
6	Fish and seafood	48.0	7.8	83.4
7	Poultry	27.2	4.5	87.9
8	Color-Green-Fruit	15.2	2.5	90.3
9	Clarified Butter and Animal Fat	12.7	2.1	92.4
10	Organ Meat	11.4	1.9	94.3
11	Sulfur	6.5	1.1	95.3
12	Broth	5.8	1	96.3
13	Leafy	5.4	0.9	97.2
14	Nutritional yeast with vitamin B12	4.1	0.7	97.9
15	Color-Orange-Fruit	3.4	0.6	98.4
16	Color-Orange-Vegetable	2.5	0.4	98.8
17	Color-Red-Fruit	2.5	0.4	99.2
18	Color-Blue-Fruit	2.2	0.4	99.6
19	Color-White-Fruit	1.2	0.2	99.8
20	Color-Green-Vegetable	0.9	0.1	99.9
21	Color-Red-Vegetable	0.2	0	100
22	Kelp, dried powder	0.1	0	100
23	Spices	0.1	0	100
24	Ingredients (e.g., vanilla, baking soda, cocoa)	0.0	0	100

Table S3C: Saturated Fat (7-day total 236.5 g saturated fat)				
**Rank**	**Food Group**	**Total g**	**% Total**	**Cumulative %**
**1**	**Coconut milk and coconut**	**102.8**	**43.5**	**43.5**
	- Coconut milk, regular (304g)	*47*		
	- Coconut milk, lite (960g)	*53.6*		
	- Coconut, dried (4g)	2.3		

Table S3C: Saturated Fat (7-day total 236.5 g saturated fat)				
**Rank**	**Food Group**	**Total g**	**% Total**	**Cumulative %**
**2**	**Fats, oil, coconut**	**47.0**	**19.9**	**63.3**
**3**	**Beef/Pork/Lamb**	**28.2**	**11.9**	**75.3**
4	Fats, oil excluding coconut	12.6	5.3	80.6
5	Nuts and seeds	9.6	4	84.6
6	Fish and seafood	9.0	3.8	88.4
7	Clarified Butter and Animal Fat	7.9	3.4	91.8
8	Poultry	7.5	3.2	95
9	Organ Meat	3.6	1.5	96.5
10	Color-Green-Fruit	2.2	0.9	97.4
11	Broth	1.7	0.7	98.2
12	Sulfur	1.1	0.5	98.6
13	Leafy	0.6	0.3	98.9
14	Color-Orange-Vegetable	0.6	0.2	99.2
15	Nutritional yeast with vitamin B12	0.5	0.2	99.4
16	Color-Orange-Fruit	0.5	0.2	99.6
17	Color-White-Fruit	0.3	0.1	99.7
18	Color-Green-Vegetable	0.2	0.1	99.8
19	Color-Blue-Fruit	0.2	0.1	99.9
20	Color-Red-Fruit	0.2	0.1	99.9
21	Kelp, dried powder	0.1	0	100
22	Spices	0.0	0	100
23	Color-Red-Vegetable	0.0	0	100
24	Ingredients (e.g., vanilla, baking soda, cocoa)	0.0	0	100
25	Tea	0.0	0	100

Table S3D: Linoleic Acid (7-day total 81.8 g linoleic acid)				
**Rank**	**Food Group**	**Total g**	**% Total**	**Cumulative %**
**1**	**Nuts and seeds**	**39.9**	**48.8**	**48.8**
	- Almonds (145g)	*17.8*		
	- Sunflower seed butter (16g)	*1.6*		
	- Walnuts (54g)	*20.5*		
**2**	**Fats, oil excluding coconut**	**9.6**	**11.8**	**60.6**
	- Flax seed oil (9g)	*1.3*		
	- Olive oil (86g)	*8.3*		
3	Beef/Pork/Lamb	7.4	9.1	69.7
4	Fish and seafood	7.0	8.6	78.3
5	Poultry	5.1	6.3	84.6
6	Color-Green-Fruit	2.0	2.5	87.1
7	Sulfur	1.6	1.9	89
8	Coconut milk and coconut	1.3	1.6	90.6
9	Organ Meat	1.3	1.5	92.1
10	Broth	1.1	1.4	93.5
11	Fats, oil, coconut	1.0	1.2	94.7
12	Color-Orange-Vegetable	0.9	1.1	95.8
13	Color-Red-Fruit	0.8	1	96.7

Table S3D: Linoleic Acid (7-day total 81.8 g linoleic acid)				
Rank	Food Group	Total g	% Total	Cumulative %
14	Color-Orange-Fruit	0.7	0.8	97.5
15	Leafy	0.6	0.7	98.3
16	Color-Blue-Fruit	0.5	0.6	98.9
17	Color-White-Fruit	0.3	0.4	99.3
18	Clarified Butter and Animal Fat	0.3	0.4	99.7
19	Color-Green-Vegetable	0.2	0.2	99.9
20	Color-Red-Vegetable	0.1	0.1	100
21	Spices	0.0	0	100
22	Nutritional yeast with vitamin B12	0.0	0	100
23	Tea	0.0	0	100

Table S3E: Dietary Fiber (7-day total 276.3 g dietary fiber)				
Rank	Food Group	Total g	% Total	Cumulative %
**1**	**Sulfur**	**46.4**	**16.8**	**16.8**
**2**	**Color-Orange-Vegetable**	**39.7**	**14.4**	**31.2**
**3**	**Leafy**	**29.0**	**10.5**	**41.7**
**4**	**Color-Orange-Fruit**	**28.8**	**10.4**	**52.1**
5	Nuts and seeds	22.6	8.2	60.3
6	Color-Blue-Fruit	21.3	7.7	68
7	Color-Red-Fruit	21.0	7.6	75.6
8	Nutritional yeast with vitamin B12	19.3	7	82.6
9	Color-Green-Fruit	17.3	6.3	88.9
10	Color-White-Fruit	15.0	5.4	94.3
11	Coconut milk and coconut	6.8	2.5	96.8
12	Color-Green-Vegetable	4.0	1.4	98.2
13	Color-Red-Vegetable	3.9	1.4	99.6
14	Spices	0.7	0.2	99.9
15	Kelp, dried powder	0.3	0.1	100
16	Ingredients (e.g., vanilla, baking soda, cocoa)	0.1	0	100

Table S3F: Vitamin A (7-day total 24,899 mcg RAE vitamin A)				
Rank	Food Group	Total mcg	% Total	Cumulative %
**1**	**Color-Orange-Vegetable**	**9216**	**37**	**37**
**2**	**Organ Meat**	**6998**	**28.1**	**65.1**
	- Chicken Liver (176g; 6.2oz)	*6998*		
**3**	**Leafy**	**5361**	**21.5**	**86.7**
4	Sulfur	2074	8.3	95
5	Color-Orange-Fruit	438	1.8	96.7
6	Fish and seafood	310	1.2	98
7	Color-Red-Fruit	140	0.6	98.5
8	Clarified Butter and Animal Fat	108	0.4	99

Table S3F: Vitamin A (7-day total 24,899 mcg RAE vitamin A)				
**Rank**	**Food Group**	**Total mcg**	**% Total**	**Cumulative %**
9	Color-Green-Vegetable	101	0.4	99.4
10	Color-Blue-Fruit	55	0.2	99.6
11	Color-Green-Fruit	39	0.2	99.8
12	Poultry	33	0.1	99.9
13	Color-White-Fruit	13	0.1	99.9
14	Beef/Pork/Lamb	7	0	100
15	Color-Red-Vegetable	2	0	100
16	Spices	1	0	100
17	Kelp, dried powder	1	0	100
18	Nuts and seeds	1	0	100

Table S3G: Retinol (7-day total 7447 mcg retinol)				
**Rank**	**Food Group**	**Total mcg**	**% Total**	**Cumulative %**
**1**	**Organ Meat**	**6992**	**93.9**	**93.9**
	- Chicken Liver (176g; 6.2oz)	*6992*		
2	Fish and seafood	310	4.2	98.1
3	Clarified Butter and Animal Fat	106	1.4	99.5
4	Poultry	33	0.4	99.9
5	Beef/Pork/Lamb	7	0.1	100

Table S3H: Vitamin C (7-day total 2552 mg vitamin C)				
**Rank**	**Food Group**	**Total mg**	**% Total**	**Cumulative %**
**1**	**Sulfur**	**754**	**29.6**	**29.6**
**2**	**Color-Orange-Fruit**	**587**	**23**	**52.6**
**3**	**Leafy**	**380**	**14.9**	**67.5**
**4**	**Color-Green-Fruit**	**252**	**9.9**	**77.3**
5	Color-Red-Fruit	203	7.9	85.3
6	Color-Orange-Vegetable	179	7	92.3
7	Color-Blue-Fruit	59	2.3	94.6
8	Organ Meat	49	1.9	96.5
9	Color-White-Fruit	33	1.3	97.8
10	Color-Green-Vegetable	31	1.2	99
11	Coconut milk and coconut	8	0.3	99.3
12	Color-Red-Vegetable	7	0.3	99.6
13	Fish and seafood	6	0.2	99.8
14	Broth	3	0.1	99.9
15	Nuts and seeds	1	0	99.9
16	Kelp, dried powder	1	0	100
17	Spices	0	0	100
18	Beef/Pork/Lamb	0	0	100

Table S3H: Vitamin C (7-day total 2552 mg vitamin C)				
**Rank**	**Food Group**	**Total mg**	**% Total**	**Cumulative %**
19	Vinegar	0	0	100

Table S3I: Vitamin D (7-day total 86 mcg vitamin D)				
**Rank**	**Food Group**	**Total mcg**	**% Total**	**Cumulative %**
**1**	**Fish and seafood**	**81**	**94**	**94**
	- Salmon (11 oz)	*71*	*83*	
	- Sardines (6.6 oz)	*9*	*11*	
	- Oysters (2.0 oz)	*0*	*0*	
2	Beef/Pork/Lamb	4	4.3	98.3
	- Pork (9.4 oz)	*2*	*2.2*	
	- Bratwurst (2.6 oz)	*1*	*1*	
	- Lamb (4.8 oz)	*0*	*0.9*	
	- Beef (3.3 oz)	*0*	*0.2*	
3	Sulfur	1	1.1	99.4
4	Poultry	0	0.4	99.8
5	Broth	0	0.2	100
6	Clarified Butter and Animal Fat	0	0	100

Table S3J: (7-day total 123 mg vitamin E)				
**Rank**	**Food Group**	**Total mg**	**% Total**	**Cumulative %**
**1**	**Nuts and seeds**	**41**	**33.6**	**33.6**
	- Almonds (145g)	*37*		
	- Sunflower seed butter (16g)	*4*		
	- Walnuts (54g)	*0*		
**2**	**Leafy**	**13**	**10.8**	**44.4**
**3**	**Fats, oil excluding coconut**	**12**	**10**	**54.4**
	- Flax seed oil (9g)	*0*		
	- Olive oil (86g)	*12*		
4	Color-Orange-Vegetable	10	8.5	63
5	Sulfur	10	7.9	70.9
6	Fish and seafood	10	7.9	78.8
7	Color-Orange-Fruit	7	6.1	84.9
8	Color-Green-Fruit	6	4.6	89.5
9	Color-Blue-Fruit	3	2.8	92.3
10	Color-Red-Fruit	2	1.9	94.2
11	Beef/Pork/Lamb	2	1.6	95.8
12	Organ Meat	1	1.2	97
13	Color-White-Fruit	1	0.8	97.8
14	Poultry	1	0.8	98.6
15	Color-Green-Vegetable	0	0.4	99

Table S3J: (7-day total 123 mg vitamin E)				
Rank	Food Group	Total mg	% Total	Cumulative %
16	Clarified Butter and Animal Fat	0	0.3	99.3
17	Coconut milk and coconut	0	0.3	99.5
18	Kelp, dried powder	0	0.2	99.7
19	Broth	0	0.1	99.8
20	Spices	0	0.1	99.9
21	Fats, oil, coconut	0	0.1	100
22	Color-Red-Vegetable	0	0	100

Table S3K: Vitamin K (7-day total 6623 mg vitamin K)				
Rank	Food Group	Total mg	% Total	Cumulative %
**1**	**Leafy**	**5133**	**77.5**	**77.5**
**2**	**Sulfur**	**985**	**14.9**	**92.4**
3	Color-Green-Fruit	150	2.3	94.7
4	Color-Blue-Fruit	99	1.5	96.1
5	Color-Orange-Vegetable	53	0.8	96.9
6	Fats, oil excluding coconut	52	0.8	97.7
7	Color-Green-Vegetable	45	0.7	98.4
8	Color-Orange-Fruit	28	0.4	98.8
9	Color-Red-Fruit	21	0.3	99.2
10	Kelp, dried powder	15	0.2	99.4
11	Color-White-Fruit	13	0.2	99.6
12	Beef/Pork/Lamb	9	0.1	99.7
13	Fish and seafood	7	0.1	99.8
14	Coconut milk and coconut	4	0.1	99.9
15	Poultry	3	0.1	99.9
16	Spices	2	0	100
17	Nuts and seeds	1	0	100
18	Clarified Butter and Animal Fat	1	0	100
19	Fats, oil, coconut	0	0	100
20	Color-Red-Vegetable	0	0	100
21	Ingredients (e.g., vanilla, baking soda, cocoa)	0	0	100

Table S3L: Vitamin B1 (7-day total 57.0 mg vitamin B1)				
Rank	Food Group	Total mg	% Total	Cumulative %
**1**	**Nutritional yeast with vitamin B12**	**47.6**	**83.6**	**83.6**
	- Nutritional Yeast (79g; 9 tablespoons)	*47.6*		
**2**	**Beef/Pork/Lamb**	**2.3**	**4**	**87.5**
	- Pork (9.4 oz)	*1.8*		
	- Bratwurst (2.6 oz)	*0.3*		
	- Lamb (4.8 oz)	*0.2*		

Table S3L: Vitamin B1 (7-day total 57.0 mg vitamin B1)				
**Rank**	**Food Group**	**Total mg**	**% Total**	**Cumulative %**
	- Beef (3.3 oz)	*0.1*		
3	Sulfur	1.4	2.4	89.9
4	Color-Orange-Vegetable	0.9	1.7	91.6
5	Color-Orange-Fruit	0.9	1.6	93.2
6	Leafy	0.8	1.5	94.7
7	Color-Green-Fruit	0.6	1	95.7
8	Organ Meat	0.5	0.9	96.6
9	Nuts and seeds	0.5	0.9	97.5
10	Fish and seafood	0.3	0.6	98
11	Color-Red-Fruit	0.3	0.5	98.5
12	Color-Blue-Fruit	0.2	0.4	98.9
13	Poultry	0.1	0.3	99.2
14	Coconut milk and coconut	0.1	0.2	99.4
15	Color-White-Fruit	0.1	0.2	99.6
16	Color-Green-Vegetable	0.1	0.2	99.8
17	Broth	0.1	0.1	99.9
18	Color-Red-Vegetable	0.0	0.1	100
19	Kelp, dried powder	0.0	0	100
20	Sweeteners	0.0	0	100
21	Spices	0.0	0	100

Table S3M: Vitamin B2 (7-day total 63.2 mg vitamin B2)				
**Rank**	**Food Group**	**Total mg**	**% Total**	**Cumulative %**
**1**	**Nutritional yeast with vitamin B12**	**47.6**	**75.3**	**75.3**
	- Nutritional Yeast (79g; 9 tablespoons)	*47.6*		
**2**	**Organ Meat**	**3.5**	**5.5**	**80.9**
	- Chicken Liver (176g; 6.2oz)	*3.5*		
3	Sulfur	2.7	4.3	85.2
4	Nuts and seeds	1.8	2.8	87.9
5	Beef/Pork/Lamb	1.5	2.4	90.3
6	Leafy	1.3	2.1	92.4
7	Fish and seafood	1.1	1.7	94.1
8	Color-Orange-Vegetable	0.9	1.4	95.5
9	Color-Orange-Fruit	0.6	0.9	96.4
10	Color-Green-Fruit	0.5	0.8	97.2
11	Poultry	0.4	0.7	97.9
12	Color-Blue-Fruit	0.3	0.5	98.4
13	Color-Red-Fruit	0.2	0.4	98.8
14	Color-White-Fruit	0.2	0.4	99.2
15	Broth	0.2	0.3	99.5
16	Color-Green-Vegetable	0.2	0.2	99.7
17	Color-Red-Vegetable	0.1	0.1	99.8
18	Tea	0.1	0.1	99.9

Table S3M: Vitamin B2 (7-day total 63.2 mg vitamin B2)				
Rank	Food Group	Total mg	% Total	Cumulative %
19	Kelp, dried powder	0.0	0.1	100
20	Sweeteners	0.0	0	100
21	Coconut milk and coconut	0.0	0	100
22	Spices	0.0	0	100
23	Ingredients (e.g., vanilla, baking soda, cocoa)	0.0	0	100

Table S3N: Vitamin B3 (7-day total 477 mg vitamin B3)				
Rank	Food Group	Total mg	% Total	Cumulative %
**1**	**Nutritional yeast with vitamin B12**	**278**	**58.2**	**58.2**
	- Nutritional Yeast (79g; 9 tablespoons)	*278*		
**2**	**Fish and seafood**	**38**	**7.9**	**66.1**
**3**	**Beef/Pork/Lamb**	**33**	**7**	**73.1**
**4**	**Sulfur**	**26**	**5.5**	**78.6**
5	Poultry	20	4.1	82.7
6	Organ Meat	19	4.1	86.8
	- Chicken Liver (176g; 6.2oz)	*19*		
7	Color-Orange-Vegetable	13	2.8	89.6
8	Color-Orange-Fruit	10	2.1	91.7
9	Broth	8	1.6	93.3
10	Leafy	7	1.5	94.8
11	Nuts and seeds	7	1.5	96.2
12	Color-Green-Fruit	5	1	97.2
13	Color-Blue-Fruit	4	0.8	98
14	Coconut milk and coconut	3	0.7	98.7
15	Color-Red-Fruit	3	0.5	99.2
16	Color-White-Fruit	2	0.4	99.6
17	Color-Green-Vegetable	1	0.3	99.8
18	Color-Red-Vegetable	0	0.1	99.9
19	Kelp, dried powder	0	0	100
20	Sweeteners	0	0	100
21	Spices	0	0	100
22	Ingredients (e.g., vanilla, baking soda, cocoa)	0	0	100

Table S3O: vitamin B6 (7-day total 66.8 mg vitamin B6)				
Rank	Food Group	Total mg	% Total	Cumulative %
**1**	**Nutritional yeast with vitamin B12**	**47.6**	**71.3**	**71.3**
	- Nutritional Yeast (79g; 9 tablespoons)	*47.6*		
**2**	**Sulfur**	**4.3**	**6.5**	**77.8**
**3**	**Color-Orange-Vegetable**	**2.5**	**3.8**	**81.5**
**4**	**Beef/Pork/Lamb**	**1.8**	**2.6**	**84.2**
**5**	**Leafy**	**1.6**	**2.4**	**86.5**
**6**	**Fish and seafood**	**1.5**	**2.2**	**88.7**

Table S3O: vitamin B6 (7-day total 66.8 mg vitamin B6)				
**Rank**	**Food Group**	**Total mg**	**% Total**	**Cumulative %**
7	Organ Meat	1.3	2	90.7
	- Chicken Liver (176g; 6.2oz)	*1.3*		
8	Color-Orange-Fruit	1.3	1.9	92.6
9	Color-Green-Fruit	1.1	1.6	94.2
10	Color-White-Fruit	0.8	1.3	95.5
11	Poultry	0.8	1.2	96.7
12	Nuts and seeds	0.6	0.9	97.6
13	Color-Blue-Fruit	0.4	0.7	98.3
14	Color-Red-Fruit	0.4	0.6	98.9
15	Color-Green-Vegetable	0.3	0.4	99.3
16	Coconut milk and coconut	0.2	0.3	99.6
17	Broth	0.1	0.2	99.7
18	Color-Red-Vegetable	0.1	0.1	99.9
19	Sweeteners	0.1	0.1	100
20	Spices	0.0	0	100

Table S3P: Folate (7-day total 6617 mcg DFE folate)				
**Rank**	**Food Group**	**Total mcg DFE**	**% Total**	**Cumulative %**
**1**	**Leafy**	**1478**	**22.3**	**22.3**
**2**	**Sulfur**	**1266**	**19.1**	**41.5**
**3**	**Nutritional yeast with vitamin B12**	**1190**	**18**	**59.4**
	- Nutritional Yeast (79g; 9 tablespoons)	*1190*		
**4**	**Organ Meat**	**1016**	**15.4**	**74.8**
	- Chicken Liver (176g; 6.2oz)	*1016*		
5	Color-Orange-Fruit	346	5.2	80
6	Color-Orange-Vegetable	200	3	83.1
7	Color-Green-Fruit	198	3	86
8	Nuts and seeds	154	2.3	88.4
9	Color-Red-Vegetable	151	2.3	90.7
10	Color-Red-Fruit	103	1.6	92.2
11	Color-Green-Vegetable	88	1.3	93.6
12	Fish and seafood	82	1.2	94.8
13	Coconut milk and coconut	76	1.1	95.9
14	Color-Blue-Fruit	60	0.9	96.8
15	Color-White-Fruit	57	0.9	97.7
16	Beef/Pork/Lamb	51	0.8	98.5
17	Kelp, dried powder	40	0.6	99.1
18	Broth	28	0.4	99.5
19	Tea	18	0.3	99.8
20	Poultry	13	0.2	100
21	Spices	2	0	100
22	Sweeteners	0	0	100

Table S3P: Folate (7-day total 6617 mcg DFE folate)				
Rank	Food Group	Total mcg DFE	% Total	Cumulative %
23	Ingredients (e.g., vanilla, baking soda, cocoa)	0	0	100

Table S3Q: Vitamin B12 (7-day total 118.8 mcg vitamin B12)				
Rank	Food Group	Total mcg	% Total	Cumulative %
**1**	**Fish and seafood**	**39.9**	**33.6**	**33.6**
	- Salmon (11 oz)	*13.2*		
	- Sardines (6.6 oz)	*16.8*		
	- Oysters (2.0 oz)	*9.9*		
**2**	**Nutritional yeast with vitamin B12**	**39.7**	**33.4**	**67**
	- Nutritional Yeast (79g; 9 tablespoons)	*39.7*		
**3**	**Organ Meat**	**29.6**	**24.9**	**91.9**
	- Chicken Liver (176g; 6.2oz)	*29.6*		
4	Beef/Pork/Lamb	7.9	6.6	98.5
5	Broth	1.0	0.8	99.4
6	Poultry	0.7	0.6	100
7	Sulfur	0.0	0	100
8	Clarified Butter and Animal Fat	0.0	0	100

Table S3R: Choline (7-day total 3120 mg choline)				
Rank	Food Group	Total mg	% Total	Cumulative %
**1**	**Beef/Pork/Lamb**	**518**	**16.6**	**16.6**
	- Pork (9.4 oz)	*231*		
	- Bratwurst (2.6 oz)	*41*		
	- Lamb (4.8 oz)	*136*		
	- Beef (3.3 oz)	*109*		
**2**	**Fish and seafood**	**510**	**16.4**	**32.9**
	- Salmon (11 oz)	*295*		
	- Sardines (6.6 oz)	*141*		
	- Oysters (2.0 oz)	*74*		
**3**	**Organ Meat**	**510**	**16.3**	**49.3**
	- Chicken Liver (176g; 6.2oz)	*510*		
**4**	**Sulfur**	**429**	**13.8**	**63**
5	Poultry	189	6.1	69.1
6	Leafy	149	4.8	73.9
7	Color-Orange-Vegetable	144	4.6	78.5
8	Color-Orange-Fruit	114	3.7	82.2
9	Nuts and seeds	106	3.4	85.6
10	Nutritional yeast with vitamin B12	105	3.4	88.9
11	Color-Green-Fruit	81	2.6	91.5

Table S3R: Choline (7-day total 3120 mg choline)				
12	Color-Red-Fruit	59	1.9	93.4
13	Coconut milk and coconut	42	1.4	94.8
14	Color-Blue-Fruit	39	1.2	96
15	Broth	38	1.2	97.2
16	Color-White-Fruit	34	1.1	98.3
17	Color-Green-Vegetable	26	0.8	99.2
18	Color-Red-Vegetable	8	0.3	99.4
19	Vinegar	7	0.2	99.7
20	Clarified Butter and Animal Fat	3	0.1	99.8
21	Kelp, dried powder	3	0.1	99.9
22	Spices	2	0.1	99.9
23	Tea	1	0	100
24	Sweeteners	1	0	100
25	Fats, oil excluding coconut	0	0	100
26	Fats, oil, coconut	0	0	100
27	Ingredients (e.g., vanilla, baking soda, cocoa)	0	0	100

Table S3S: Betaine (7-day total 938 mg betaine)

Rank	Food Group	Total mg	% Total	Cumulative %
**1**	**Leafy**	**325**	**34.7**	**34.7**
**2**	**Color-Red-Vegetable**	**178**	**19**	**53.7**
**3**	**Color-Orange-Vegetable**	**158**	**16.9**	**70.6**
4	Beef/Pork/Lamb	76	8.1	78.7
5	Nutritional yeast with vitamin B12	53	5.6	84.3
6	Sulfur	43	4.6	88.9
7	Fish and seafood	41	4.3	93.2
8	Organ Meat	22	2.4	95.6
9	Poultry	15	1.6	97.2
10	Nuts and seeds	7	0.7	97.9
11	Tea	4	0.4	98.3
12	Broth	3	0.4	98.7
13	Color-Red-Fruit	3	0.3	99
14	Color-Orange-Fruit	3	0.3	99.3
15	Color-Green-Fruit	2	0.3	99.5
16	Color-Blue-Fruit	1	0.2	99.7
17	Coconut milk and coconut	1	0.1	99.8
18	Color-White-Fruit	1	0.1	99.8
19	Color-Green-Vegetable	1	0.1	99.9
20	Vinegar	0	0	100
21	Spices	0	0	100
22	Fats, oil excluding coconut	0	0	100
23	Sweeteners	0	0	100
24	Kelp, dried powder	0	0	100
25	Clarified Butter and Animal Fat	0	0	100

Table S3S: Betaine (7-day total 938 mg betaine)				
Rank	Food Group	Total mg	% Total	Cumulative %
26	Ingredients (e.g., vanilla, baking soda, cocoa)	0	0	100

Table S3T: Beta-Carotene (7-day total 195,808 mcg beta-carotene)				
Rank	Food Group	Total mcg	% Total	Cumulative %
**1**	**Color-Orange-Vegetable**	**97827**	**50**	**50**
**2**	**Leafy**	**64168**	**32.8**	**82.7**
**3**	**Sulfur**	**24884**	**12.7**	**95.4**
4	Color-Orange-Fruit	4831	2.5	97.9
5	Color-Red-Fruit	1571	0.8	98.7
6	Color-Green-Vegetable	1197	0.6	99.3
7	Color-Blue-Fruit	612	0.3	99.6
8	Color-Green-Fruit	445	0.2	99.9
9	Color-White-Fruit	124	0.1	99.9
10	Organ Meat	53	0	100
11	Color-Red-Vegetable	28	0	100
12	Clarified Butter and Animal Fat	25	0	100
13	Kelp, dried powder	16	0	100
14	Spices	15	0	100
15	Nuts and seeds	13	0	100

Table S3U: Beta-Cryptoxanthin (7-day total 10,687 mcg beta-cryptoxanthin)				
Rank	Food Group	Total mcg	% Total	Cumulative %
**1**	**Color-Orange-Vegetable**	**9363**	**87.6**	**87.6**
	- Carrots (319g)	*0*		
	- Squash, winter (505g)	*9363*		
	- Squash, summer (57g)	*0*		
	- Sweet potato/yam (463g)	*0*		
**2**	**Color-Orange-Fruit**	**768**	**7.2**	**94.8**
**3**	**Leafy**	**209**	**2**	**96.8**
**4**	**Color-Red-Fruit**	**185**	**1.7**	**98.5**
5	Color-Blue-Fruit	82	0.8	99.2
6	Color-White-Fruit	24	0.2	99.5
7	Color-Green-Fruit	23	0.2	99.7
8	Organ Meat	19	0.2	99.9
9	Color-Green-Vegetable	14	0.1	100
10	Spices	1	0	100

Table S3V: Lutein + Zeaxanthin (7-day total 98,704 mcg lutein + zeaxanthin)				
**Rank**	**Food Group**	**Total mcg**	**% Total**	**Cumulative %**
**1**	**Leafy**	**82331**	**83.4**	**83.4**
2	Sulfur	5240	5.3	88.7
3	Color-Orange-Vegetable	5119	5.2	93.9
4	Color-Green-Vegetable	2933	3	96.9
5	Color-Orange-Fruit	993	1	97.9
6	Color-Green-Fruit	817	0.8	98.7
7	Color-Blue-Fruit	490	0.5	99.2
8	Color-Red-Fruit	445	0.5	99.7
9	Color-White-Fruit	174	0.2	99.8
10	Organ Meat	146	0.1	100
11	Spices	10	0	100
12	Nuts and seeds	6	0	100
13	Ingredients (e.g., vanilla, baking soda, cocoa)	0	0	100

Table S3W: Lycopene (7-day total 12,038 mcg lycopene)				
**Rank**	**Food Group**	**Total mcg**	**% Total**	**Cumulative %**
**1**	**Color-Red-Fruit**	**11,965**	**99.4**	**99.4**
	- Cherries (154g)	*0*		
	- Pink grapefruit (115g)	*1632*		
	- Raspberries (185g)	*0*		
	- Strawberries (152g)	*0*		
	- Watermelon (228g)	*10333*		
2	Organ Meat	37	0.3	99.7
3	Sulfur	27	0.2	99.9
4	Color-Orange-Fruit	7	0.1	100
5	Color-Orange-Vegetable	3	0	100
6	Spices	0	0	100

Table S3X: Calcium (7-day total 5707 mg calcium)				
**Rank**	**Food Group**	**Total g**	**% Total**	**Cumulative %**
**1**	**Sulfur**	**1290**	**22.6**	**22.6**
	- Mushrooms (409g)	*19*		
	- Broccoli (354g)	*142*		
	- Cabbage (232g)	*83*		
	- Onions (282g)	*63*		
	- Bok Choy (785g)	*814*		
	- Asparagus (90g)	*21*		
	- Turnips (217g)	*68*		
	- Garlic (32.5g)	*59*		
	- Chives (3g)	*3*		
	- Cauliflower(102g)	*20*		

Table S3X: Calcium (7-day total 5707 mg calcium)				
**Rank**	**Food Group**	**Total g**	**% Total**	**Cumulative %**
**2**	**Fish and seafood**	**1176**	**20.6**	**43.2**
	- Salmon (11 oz)	390		
	- Sardines (6.6 oz)	720		
	- Oysters (2.0 oz)	66		
**3**	**Leafy**	**1048**	**18.4**	**61.6**
	- Kale (114g)	*157*		
	- Spinach (315g)	*312*		
	- Mustard greens (132g)	*155*		
	- Romaine (400g)	*132*		
	- Cilantro (28g)	*19*		
	- Parsley (65g)	*90*		
	- Collards (107g)	*183*		
4	Nuts and seeds	452	7.9	69.5
5	Color-Orange-Vegetable	449	7.9	77.4
6	Color-Orange-Fruit	289	5.1	82.4
7	Color-Green-Fruit	144	2.5	84.9
8	Color-Red-Fruit	132	2.3	87.2
9	Color-Blue-Fruit	117	2	89.3
10	Color-Green-Vegetable	79	1.4	90.7
11	Sweeteners	67	1.2	91.9
12	Kelp, dried powder	62	1.1	92.9
13	Nutritional yeast with vitamin B12	56	1	93.9
14	Broth	50	0.9	94.8
15	Vinegar	49	0.9	95.7
16	Poultry	49	0.9	96.5
17	Beef/Pork/Lamb	47	0.8	97.3
18	Coconut milk and coconut	46	0.8	98.1
19	Color-White-Fruit	37	0.6	98.8
20	Color-Red-Vegetable	22	0.4	99.2
21	Organ Meat	19	0.3	99.5
22	Spices	13	0.2	99.7
23	Salt	13	0.2	100
24	Fats, oil excluding coconut	1	0	100
25	Ingredients (e.g., vanilla, baking soda, cocoa)	1	0	100
26	Fats, oil, coconut	1	0	100
27	Clarified Butter and Animal Fat	1	0	100

Table S3Y: Copper (7-day total 21.2 mg copper)				
**Rank**	**Food Group**	**Total mg**	**% Total**	**Cumulative %**
**1**	**Fish and seafood**	**3.8**	**18**	**18**
	- Oysters (57g)	3.2		
**2**	**Sulfur**	**2.8**	**13**	**31**
	- Mushrooms (409g)	1.6		
	- Broccoli (354g)	0.2		
	- Cabbage (232g)	0.2		
	- Onions (282g)	0.2		

Table S3Y: Copper (7-day total 21.2 mg copper)				
**Rank**	**Food Group**	**Total mg**	**% Total**	**Cumulative %**
	- Bok Choy (785g)	0.2		
	- Asparagus (90g)	0.1		
	- Turnips (217g)	0.1		
	- Garlic (32.5g)	0.1		
	- Other[2]	0.0		
**3**	**Nuts and seeds**	**2.6**	**12.3**	**43.3**
	- Almonds (145g)	1.5		
	- Walnuts (54g)	0.9		
	- Sunflower butter (16g)	0.3		
**4**	**Leafy**	**2.5**	**11.7**	**54.9**
	- Kale (114g)	1.5		
	- Spinach (315g)	0.4		
	- Mustard greens (132g)	0.2		
	- Romaine (400g)	0.2		
	- Cilantro (28g)	0.1		
	- Parsley (65g)	0.1		
	- Collards (107g)	0.1		
5	Color-Orange-Fruit	1.4	6.6	61.6
6	Coconut milk and coconut	1.3	6.1	67.7
7	Color-Orange-Vegetable	1.2	5.8	73.4
8	Color-Green-Fruit	1.0	4.8	78.3
9	Organ Meat	0.9	4.1	82.4
10	Color-Blue-Fruit	0.8	3.7	86
11	Nutritional yeast with vitamin B12	0.6	3	89.1
12	Beef/Pork/Lamb	0.5	2.5	91.5
13	Color-Red-Fruit	0.5	2.2	93.7
14	Color-White-Fruit	0.3	1.6	95.3
15	Broth	0.3	1.3	96.7
16	Poultry	0.2	0.8	97.5
17	Color-Green-Vegetable	0.2	0.7	98.2
18	Sweeteners	0.1	0.7	98.8
19	Color-Red-Vegetable	0.1	0.5	99.3
20	Vinegar	0.0	0.2	99.6
21	Tea	0.0	0.2	99.7
22	Kelp, dried powder	0.0	0.1	99.9
23	Spices	0.0	0.1	100
24	Ingredients (e.g., vanilla, baking soda, cocoa)	0.0	0	100

Table S3Z: Iron (7-day total 133.1 mg iron)				
**Rank**	**Food Group**	**Total mg**	**% Total**	**Cumulative %**
**1**	**Leafy**	**20.6**	**15.5**	**15.5**
	- Collards (107g)	*1.0*		
	- Cilantro (28g)	*0.5*		
	- Kale (114g)	*1.6*		
	- Romaine (400g)	*3.9*		
	- Mustard greens (132g)	*1.1*		
	- Parsley (65g)	*4.0*		
	- Spinach (315g)	*8.5*		

Table S3Z: Iron (7-day total 133.1 mg iron)				
**Rank**	**Food Group**	**Total mg**	**% Total**	**Cumulative %**
**2**	**Organ Meat**	**20.4**	**15.4**	**30.8**
	- Chicken Liver (176g; 6.2 oz)	*20.4*		
**3**	**Sulfur**	**19.2**	**14.4**	**45.3**
**4**	**Fish and seafood**	**13.1**	**9.9**	**55.1**
5	Beef/Pork/Lamb	8.9	6.7	61.8
6	Nuts and seeds	7.6	5.7	67.5
7	Color-Orange-Vegetable	7.1	5.3	72.9
8	Coconut milk and coconut	6.2	4.6	77.5
9	Color-Orange-Fruit	4.1	3.1	80.6
10	Nutritional yeast with vitamin B12	3.8	2.9	83.4
11	Color-Blue-Fruit	3.5	2.6	86
12	Color-Green-Fruit	3.3	2.5	88.5
13	Color-Red-Fruit	3.1	2.3	90.8
14	Poultry	3.1	2.3	93.1
15	Broth	2.6	2	95.1
16	Vinegar	1.3	1	96.1
17	Color-Red-Vegetable	1.1	0.8	96.9
18	Color-White-Fruit	1.0	0.8	97.7
19	Color-Green-Vegetable	1.0	0.8	98.5
20	Kelp, dried powder	0.9	0.7	99.2
21	Fats, oil excluding coconut	0.5	0.4	99.5
22	Sweeteners	0.2	0.2	99.7
23	Spices	0.2	0.2	99.9
24	Tea	0.1	0.1	99.9
25	Ingredients (e.g., vanilla, baking soda, cocoa)	0.0	0	100
26	Fats, oil, coconut	0.0	0	100
27	Salt	0.0	0	100

Table S3AA: Magnesium (7-day total 3191 mg magnesium)				
**Rank**	**Food Group**	**Total mg**	**% Total**	**Cumulative %**
**1**	**Nuts and seeds**	**526**	**16.5**	**16.5**
**2**	**Leafy**	**435**	**13.6**	**30.1**
**3**	**Sulfur**	**388**	**12.2**	**42.3**
4	Fish and seafood	296	9.3	51.5
5	Color-Orange-Vegetable	280	8.8	60.3
6	Color-Orange-Fruit	179	5.6	65.9
7	Coconut milk and coconut	137	4.3	70.2
8	Beef/Pork/Lamb	126	3.9	74.2
9	Color-Green-Fruit	125	3.9	78.1
10	Color-Red-Fruit	110	3.5	81.6
11	Nutritional yeast with vitamin B12	103	3.2	84.8
12	Color-Blue-Fruit	98	3.1	87.9
13	Color-White-Fruit	74	2.3	90.2

Table S3AA: Magnesium (7-day total 3191 mg magnesium)				
Rank	Food Group	Total mg	% Total	Cumulative %
14	Poultry	58	1.8	92
15	Color-Green-Vegetable	52	1.6	93.6
16	Organ Meat	44	1.4	95
17	Sweeteners	33	1	96
18	Color-Red-Vegetable	32	1	97
19	Kelp, dried powder	31	1	98
20	Broth	23	0.7	98.7
21	Vinegar	23	0.7	99.4
22	Tea	11	0.3	99.8
23	Spices	4	0.1	99.9
24	Ingredients (e.g., vanilla, baking soda, cocoa)	1	0	100
25	Salt	1	0	100

Table S3AB: Potassium (7-day total 27,117 mg potassium)				
Rank	Food Group	Total mg	% Total	Cumulative %
**1**	**Sulfur**	**6426**	**17.3**	**17.3**
	- Mushrooms (409g)	*1380*		
	- Broccoli (354g)	*1037*		
	- Cabbage (232g)	*499*		
	- Onions (282g)	*458*		
	- Bok Choy (785g)	*2079*		
	- Asparagus (90g)	*202*		
	- Turnips (217g)	*403*		
	- Garlic (32.5g)	*130*		
	- Chives (3g)	*9*		
	- Cauliflower(102g)	*229*		
**2**	**Color-Orange-Vegetable**	**4510**	**12.1**	**29.5**
	- Carrots (319g)	*979*		
	- Winter squash (505g)	*1347*		
	- Summer squash (57g)	*148*		
	- Sweet potato/yam (463g)	*2035*		
**3**	**Leafy**	**4136**	**11.1**	**40.6**
**4**	**Color-Orange-Fruit**	**2960**	**8**	**48.6**
**5**	**Color-Green-Fruit**	**2667**	**7.2**	**55.8**
6	Fish and seafood	2149	5.8	61.6
7	Beef/Pork/Lamb	1929	5.2	66.8
8	Nutritional yeast with vitamin B12	1586	4.3	71
9	Color-Blue-Fruit	1581	4.3	75.3
10	Nuts and seeds	1391	3.7	79
11	Broth	1297	3.5	82.5
12	Color-Red-Fruit	1264	3.4	85.9
13	Coconut milk and coconut	1179	3.2	89.1
14	Color-White-Fruit	1092	2.9	92
15	Color-Green-Vegetable	812	2.2	94.2

Table S3AB: Potassium (7-day total 27,117 mg potassium)				
**Rank**	**Food Group**	**Total mg**	**% Total**	**Cumulative %**
16	Poultry	548	1.5	95.7
17	Organ Meat	462	1.2	97
18	Color-Red-Vegetable	451	1.2	98.2
19	Vinegar	226	0.6	98.8
20	Sweeteners	150	0.4	99.2
21	Tea	131	0.4	99.5
22	Kelp, dried powder	124	0.3	99.9
23	Spices	35	0.1	100
24	Ingredients (e.g., vanilla, baking soda, cocoa)	10	0	100
25	Fats, oil excluding coconut	1	0	100
26	Clarified Butter and Animal Fat	1	0	100
27	Salt	0	0	100

Table S3AC: Sodium (7-day total 16,560 mg sodium)				
**Rank**	**Food Group**	**Total mg**	**% Total**	**Cumulative %**
**1**	**Salt**	**6757**	**40.8**	**40.8**
**2**	**Beef/Pork/Lamb**	**3594**	**21.7**	**62.5**
**3**	**Broth**	**1634**	**9.9**	**72.4**
4	Sulfur	1608	9.7	82.1
5	Fish and seafood	1221	7.4	89.5
6	Leafy	399	2.4	91.9
7	Color-Orange-Vegetable	390	2.4	94.2
8	Poultry	209	1.3	95.5
9	Kelp, dried powder	149	0.9	96.4
10	Organ Meat	134	0.8	97.2
11	Color-Red-Vegetable	108	0.7	97.8
12	Color-Green-Vegetable	88	0.5	98.4
13	Color-Green-Fruit	86	0.5	98.9
14	Coconut milk and coconut	44	0.3	99.2
15	Vinegar	41	0.2	99.4
16	Nutritional yeast with vitamin B12	25	0.2	99.6
17	Color-Orange-Fruit	22	0.1	99.7
18	Color-Blue-Fruit	12	0.1	99.8
19	Tea	11	0.1	99.8
20	Sweeteners	10	0.1	99.9
21	Color-Red-Fruit	6	0	99.9
22	Color-White-Fruit	6	0	100
23	Nuts and seeds	3	0	100
24	Fats, oil excluding coconut	2	0	100
25	Spices	1	0	100
26	Ingredients (e.g., vanilla, baking soda, cocoa)	0	0	100
27	Clarified Butter and Animal Fat	0	0	100

[1] Calculations were done using multiple decimal places, but results are rounded for display purposes.
